# Speed of sound for understanding metals in extreme environments

**DOI:** 10.1063/5.0186669

**Published:** 2024-12

**Authors:** Elizabeth G. Rasmussen, Boris Wilthan

**Affiliations:** National Institute of Standards and Technology, Boulder, Colorado 80305-3337, USA

## Abstract

Knowing material behavior is crucial for successful design, especially given the growing number of next-generation energy, defense, and manufacturing systems operating in extreme environments. Specific applications for materials in extreme environments include fusion energy, semiconductor manufacturing, metal additive manufacturing, and aerospace. With increased applications, awareness of foundational science for materials in extreme environments is imperative. The speed of sound provides insights into phase boundaries, like shock-induced melting. Thermodynamic integration of the speed of sound enables the deduction of other desirable properties that are difficult to measure accurately, like density, heat capacity, and expansivity. Metrology advancements enable the speed of sound to be measured at extreme conditions up to 15 000 K and 600 GPa. This comprehensive review presents state-of-the-art sound speed metrology while contextualizing it through a historical lens. Detailed discussions on new standards and metrology best practices, including uncertainty reporting, are included. Data availability for condensed matter speed of sound is presented, highlighting significant gaps in the literature. A theoretical section covers empirically based theoretical models like equations of state and CALPHAD models, the growing practice of using molecular dynamics and density functional theory simulations to fill gaps in measured data, and the use of artificial intelligence and machine learning prediction tools. Concluding, we review how a lack of measurement methods leads to gaps in data availability, which leads to data-driven theoretical models having higher uncertainty, thus limiting confidence in optimizing designs via numerical simulation for critical emerging technologies in extreme environments.

## I. INTRODUCTION

**FIG. 1.**
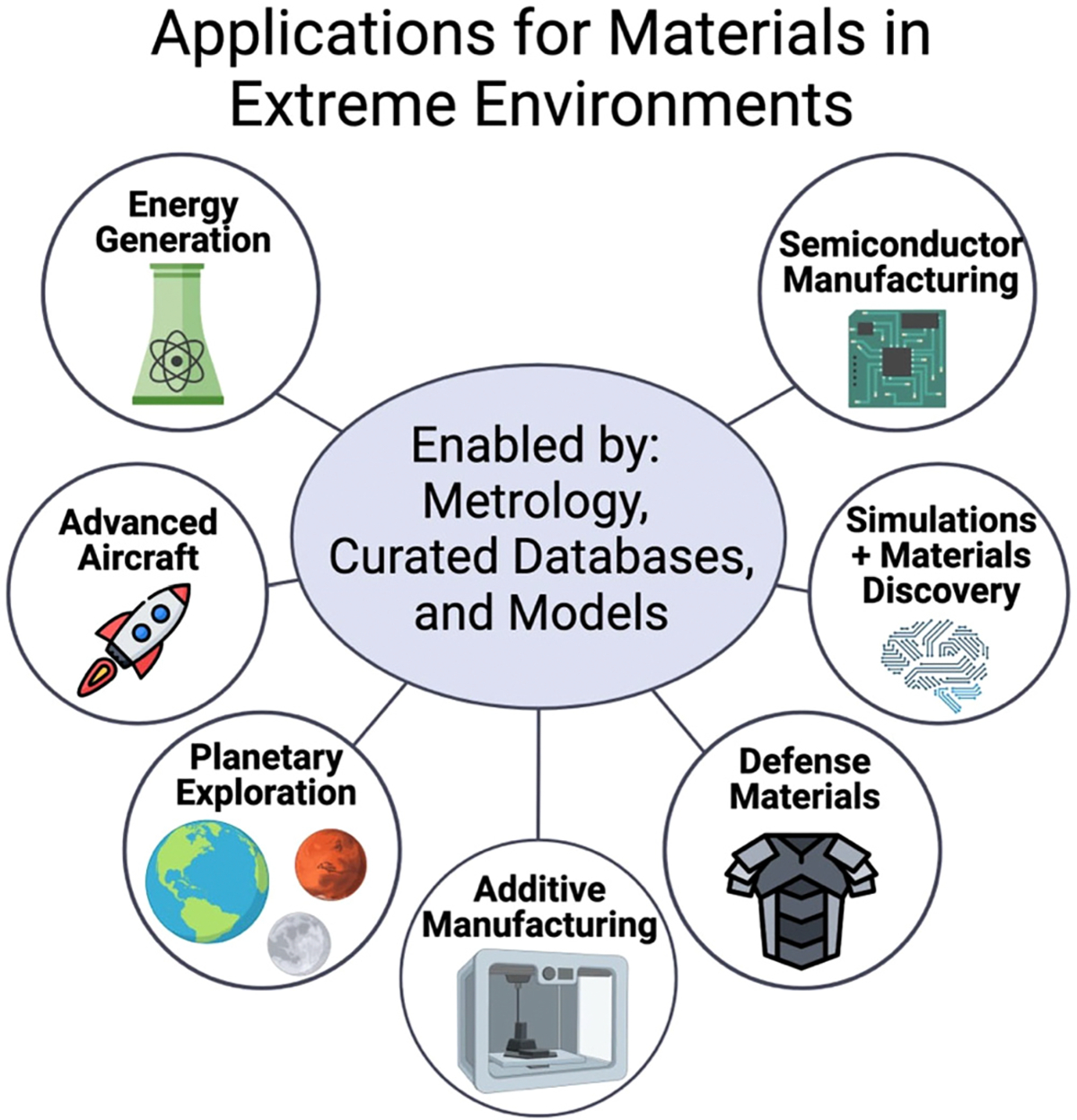
Overview of some applications for materials in extreme environments with these diverse applications centering around metrology, curated databases of measurement data, and models that rely on data from curated databases.

This paper provides a comprehensive review of the current state of the art for speed of sound (SoS) experimental techniques, data availability, theory, and applications in the solid and liquid phases at extreme conditions. The overarching theme of this review centers around metrology, or the science of measurement. Metrology is not an emerging topic but has been continuously impactful in material development and science overall in the past two decades. However, there is increased application of materials in extreme environments that rely on metrology for accurate material properties (Fig. 1). Fusion reactors for commercial clean energy exemplify a growing need for materials in extreme environments.^1^ Additionally, understanding how a material behaves under extreme pressures, like those reached in shock compression experiments, is valuable in designing non-penetrable armor and advanced aircrafts.^2^ The past 20 years have seen continued investment into additive manufacturing of metals which has applications in custom prosthetics^3,4^ and manufacturing complex shapes^5^ with the flexibility of fabrication in remote locations.^6^ Extreme environments are also a challenge in the recently deployed commercial extreme ultraviolet (EUV) lithography machines, where liquid tin is converted to a plasma by a high-power CO_2_ laser to produce a 13.5 nm wavelength light source, which allows for the smallest features to be printed on semiconductor chips.^7–9^ Beyond current applications, efforts toward discovering materials with desirable properties are being enabled by curated databases of material properties.^10^ Finally, fundamental scientific questions centered around understanding the thermodynamic properties of condensed matter in extreme environments are being explored with direct implications in geophysics and astronomy.^11^ All these applications are enabled by the advancement of metrology, the application of metrology best practices and increasingly, models that are dependent on experimental data with low uncertainty.

When reviewing applied applications for materials in extreme environments, it was found that many state-of-the-art systems rely on sparse data measured 15–80years ago by techniques that have remained largely unchanged since the 1980s and with measurement uncertainties between 4% to over 20%. This large measurement uncertainty in material properties greatly limits empirically based thermodynamic models like the Calculation of Phase Diagrams (CALPHAD) and equations of state (EoS) that define phase boundaries and material behavior.^12^ In turn, the lack of growth in first-principal physics that is foundational for the growing application space inspired the current review so that scientists are educated, and those that influence scientific funding become aware of the value of fundamental research into the thermophysical properties of materials. We hope that through this review, balance is returned to the experimental vs theoretical of scientific discovery, and experimental pursuits strive for the highest quality and best practices in metrology.

The structure of this paper is organized by how it answers the following questions:

1. What makes a material’s speed of sound a uniquely valuable thermodynamic property?
2. In the past 20 years, what is the state of innovation, continuation, or degradation for the speed of sound experimental methods?
3. Is there sufficient data availability for the speed of sound of condensed matter in extreme environments?
  - What is the current state of data availability?
  - Where are there gaps in data?
4. To what extent is the speed of sound useful in theoretical models?
5. What are the current applications and challenges of sound speed?
  - Is the application area growing or shrinking?
6. What is the outlook for the thermodynamic metrology of materials in extreme conditions?

To address these questions, this review has five sections. This first section introduces the overall review by defining the thermodynamic material property speed of sound and familiarizes the reader with the advantage speed of sound offers from both an experimental and theoretical perspective. Section II outlines the speed of sound measurement methods. There are five sub-sections in the second section: Sec. IIA covers how sound is generated in a sample and considerations when choosing a method. Section II B covers common design selections and their effect on measurement uncertainty, which is unique as we emphasize the metrology considerations for experiments and put historical and current practices for reporting experimental results into context for emerging investigators. Section IIC covers high-temperature methods (600–10 000 K), including static ultrasonic interferometric and dynamic heating methods. Section IID covers high-pressure methods (0.1–600 GPa) to measure the speed of sound of condensed matter, like using a pressure vessel, anvil press, diamond anvil cell, or shock compression. The third section covers the speed of sound data availability in the literature and has two sub-sections. Section III A covers a discussion of types of data and databases and informs the reader on historical and state-of-the-art data curation efforts globally for condensed matter materials in extreme environments. Section IIIB provides a more in-depth overview of curated databases with special emphasis on the NIST databases. Following that, we demonstrate how interconnected measurement techniques are with modeling advances in Sec. IV. Section IV covers theoretical models from sound speed measurements. We cover historical and new theoretical advancements for deriving other material properties from the speed of sound (Sec. IVA), fitting thermodynamic equation of state models (Sec. IVB), and conclude the overall section by discussing other models like DFT, MD, and ML/AI methods (Sec. IVC). Section V contains a concise presentation of applications for the speed of sound, ranging from advanced manufacturing and planetary exploration to other next-generation technologies. These underline the growing opportunity landscape for materials in extreme environments. The section concludes the paper by summarizing the article and encouraging continued development in the field.

By exerting a great effort toward reviewing in a single manuscript the experimental methods, data curation, theoretical methods, and applications for the thermodynamic metrology of the speed of sound for condensed matter in extreme environments, we hope this review encourages further research in this field to overcome some of the challenges and inspire productive research based on first-principal physics.

### A. Thermodynamic metrology: Advantages of speed of sound

**FIG. 2.**
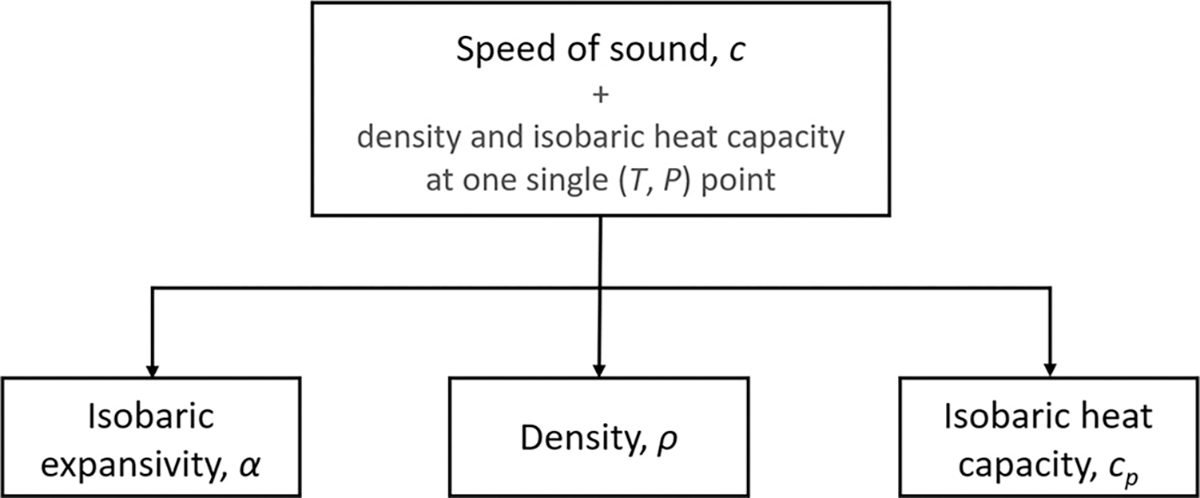
Schematic showing which other thermodynamic properties can be obtained from speed of sound data.

Thermodynamic metrology is focused on the science of measuring thermodynamic properties. Measurements of all properties under all conditions for all materials is an unrealistic task to understand the thermodynamic properties of a material. Thankfully, different thermodynamic properties have proven to be readily available to measure with low measurement uncertainty. This leads us to the thermodynamic property, speed of sound. The speed of sound (SoS) thermodynamic material property has three key advantages. First, from a metrology perspective, direct SoS measurement techniques offer the prospect of high accuracy over a wide temperature and pressure range,^13^ whereas other direct measurements, for example of density, have technical difficulties leading to much larger uncertainties.^14^ Second, experimental data on SoS for liquid materials can be used to estimate the critical temperature, as shown by Blairs and Abbasi for 36 molten metals^15^ and used later by others for elements with high-temperature melting points like tantalum.^16–18^ Third, as SoS is a second derivative property, it can be used to thermodynamically derive other thermodynamic material properties such as density, specific heat, and isothermal compressibility that are more difficult to measure accurately (see Fig. 2). Many researchers have noted the speed of sound’s derivation advantage, and the propagation of error from derivation was investigated for water by Trusler and Lemon. Their work demonstrated that speed of sound measurements, combined with density and isobaric heat capacity data at a *single* (*T*, *P*) point, can be used to accurately derive the additional thermophysical properties density, isobaric expansivity, and isobaric heat capacity at *any T* and *P* (see Fig. 2).^19^

A discussion of the speed of sound thermodynamic property derivation and modeling is covered in Sec. IV. In succession, the advantage of the speed of sound material property is apparent from both an experimental and theoretical perspective. So, when discussing metrology for thermodynamic properties, it is valuable to pay special attention to speed of sound.

### B. Scope

This review covers the thermodynamic property of the SoS at extreme conditions. Currently, there is no universal definition of extreme conditions, and it is recognized that one can define extreme conditions, or extreme environments, at either the low or high end of the chosen state variable (*T*, *P*, etc.) range. Herein the focus is on the high extremes of *T* and *P* and we define extreme environments as temperatures above 600 K with pressures from atmospheric pressure up to 600 GPa. For example, experiments at 1700 K but only atmospheric pressure as well as experiments at 200 GPa but only room temperature are considered. This specification is needed as much research ventures have focused on either extreme temperature or extreme pressure, with only the most recent works operating at extreme temperatures and pressures simultaneously. Research published from 1900 to 2022 will be covered, with an emphasis on publications from the past 20 years. Only condensed phases of matter, the solid and liquid phases, will be covered, with extra focus placed on the liquid phase. As this review focuses on the SoS for solid and liquid phases of materials, we refer the reader to work by J. P. M. Trusler on physical acoustics and metrology of materials in the gas, liquid, and near the critical point.^20^ For details specifically focused on ultrasonic techniques in fluids, we suggest work by M. Povey.^21^

**FIG. 3.**
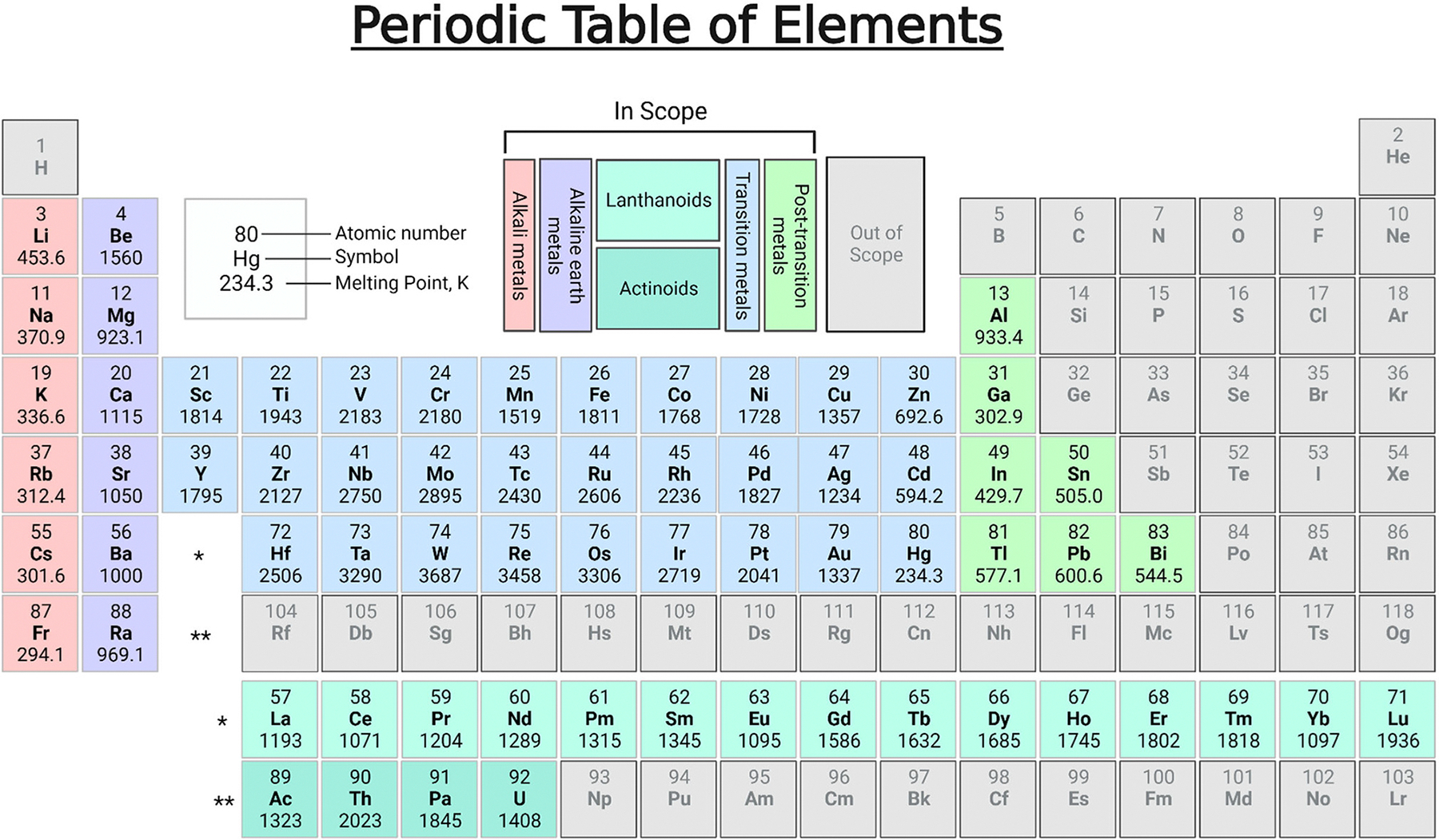
Periodic table of elements with atomic number, symbol, and melting point. Melting points defined from CRC Handbook of Chemistry and Physics, converted to the Kelvin scale and limited to four significant figures without rounding.^22^ The color indicates the classification of elements investigated or those out of scope for this review. Of the 118 elements in the periodic table, 67 are considered.

Materials composed of both multielement, and single elements will be covered, with a focus on pure elements. Regarding elements in scope for this review: while there are 118 elements defined on the periodic table of elements, this review does not consider the 17 nonmetals (noble gasses and reactive nonmetals or metalloids). In addition to the 17 nonmetals, this review will also not cover synthetic metal elements, treacly studied, or highly radioactive elements like polonium. Given these constraints, 67 elements are in scope: 6 alkali metals, 6 alkaline earth metals, 29 transition metals, 15 lanthanoids also known as “rare earth metals”, 4 actinoids, and 7 post-transition elements. Figure 3 shows the periodic table of elements and given the scope of this study is condensed matter the common atomic weight value has been replaced with the defined melting point in the Kelvin temperature scale.

This is a timely article as it updates and builds upon other reviews in the scope of our article. For example, the most recent review on data availability of sound speed data for metals was published 17 years ago by Blairs.^23^ A review by Anderson and Liebermann is also a recommended reference that discusses the transmission of sound in liquid phase metals, but as it was published over 50years ago, advances and new data are presented here.^24^ Regarding reviews that cover experimental methods in extreme environments, in 2014 Li and Liebermann published a review focusing on high-pressure experimental methods, and since publication, there have been advancements that are important to condense and summarize for readers. Boivineau and Pottlacher published a review in 2006 on high-temperature techniques using dynamic heating, but it is now over 15years old, so again an update could be useful.^26^ Additionally, this article reviews the recent theoretical and simulated models for speed of sound and the growing applications of the SoS for condensed matter materials in extreme environments. Therefore, this comprehensive review covers the outlined scope and is a reference for those emerging and established on the topic of materials in extreme environments.

## II. SPEED OF SOUND MEASUREMENT

This section describes measurement methods for the SoS of condensed matter. At the most basic level speed of sound measurement involves length and time. Propagated waves define the time interval and the sample dimensions limit the length in a setup. Thus, an SoS experiment must consider how to: (1) generate sound waves and (2) record the sound waves that pass through a sample with a (3) known length. To get a material to extreme conditions (4) temperature generation and the (5) temperature measurement as well as (6) pressure generation and (7) pressure measurement must all be carefully integrated in one experiment design.

This section contains five sub-sections meant to review and guide condensed matter SoS measurements at extreme conditions. First, an overview of sound wave generation and design considerations are presented. Second, the impact of apparatus and experiment design on the uncertainty of measurements, including a discussion on understanding a measurement technique’s limits while appreciating the value it offers. Third, high-temperature, 650–10 000 K, measurement methods at atmospheric conditions below 0.1 GPa. Fourth, high-pressure, 0.1–600 GPa, methods. When describing measurement methods the range of conditions, or even the exact conditions are listed to emphasize (a) the lower range of a technique is not necessarily at atmospheric conditions, and (b) how some techniques led to only a few (<20) data points collected per experimental report. Fifth, discussion on respecting a method’s capabilities and limits is presented. It should be noted that depending on the year of publication, the branch of science, country, and application, different nomenclature has been used to describe the same focus. To aid the reader and to decrease confusion, we use common terms seen across the literature in this review. Lists with terms used are compiled in the Nomenclature section. A list of the naming schemes for different terms is included in supplementary material.

It is hoped that by outlining apparatuses, the reader is quickly familiarized with the current state of the art and efforts toward experimental innovation are quickened. Furthermore, by including a discussion on measurement uncertainty, the reader could gain an appreciation for thoughtful design and measurement reporting integrity. Thoughtful design can aid in increasing data availability (Sec. III), theoretical modeling (Sec. IV), and applications for the speed of sound (Sec. V).

### A. Generating sound waves

When measuring the speed of sound in condensed matter materials under extreme conditions, transducers and lasers are employed in distinct ways. Transducers, typically piezoelectric devices, are utilized to generate mechanical vibrations in the material. These vibrations are generated by converting electrical energy into mechanical energy. By precisely measuring the time it takes for these vibrations to travel a known distance through the material, the speed of sound can be calculated. This method is suitable for a wide range of materials and extreme conditions, including high pressures and temperatures.

Laser-based techniques, on the other hand, utilize the principles of interferometry to measure the speed of sound. In this approach, a laser beam is directed at the material under investigation. The laser light interacts with the material, leading to the generation of reflected waves. By carefully analyzing the phase shifts and interference patterns between the incident and reflected waves, scientists can deduce the velocity of sound in the material. This method offers high precision and accuracy in determining the speed of sound under extreme conditions and is often employed when studying materials subjected to intense pressures, high temperatures, or challenging environments.

Both transducers and lasers provide valuable tools for measuring the speed of sound in condensed matter materials at extreme conditions. Their complementary approaches enable researchers to obtain precise and reliable data, contributing to a deeper understanding of the properties and behavior of materials under extreme circumstances. In this section, only the considerations for the generation of sound going through a sample will be discussed while more details on the configuration inside an apparatus capable of supporting high temperatures and high pressures are given in Secs. II C and IID, respectively.

#### 1. Transducer

A transducer’s resonance frequency is selected based on the size of the sample and material’s SoS frequency dependence. Smaller sample volumes, and thus higher resonance frequency transducers, are affiliated with techniques that operate at high pressures, and vice versa.

For low pressure ultrasonic interferometric techniques, sound waves are commonly generated by a MHz ultrasonic transducer made of Pb(Zr-Ti)O_3_ (PZT) or quartz crystal with resonance frequencies ranging from 4 MHz up to 20 MHz, although most transducers are in the 5 and 15 MHz range. PZT ultrasonic transducers are also usually x-cut so that the thickness mode of vibration occurs when the slice is electrically stimulated in the x-direction. When using a PZT crystal, it is important to report the center frequency, as PZT crystals can operate in various modes, which can be valuable for assessing deviations in sound speed measurements in viscous fluids like silicate melts or other geologically significant condensed materials.^27^ A limiting factor in the application of PZT and quartz crystals is their low Curie temperature of about 573 and 846 K, respectively.^28^ Above that temperature this material loses its spontaneous polarization and stops to function as intended but piezoelectric elements like AlN, YCOB, and LiNbO_3_ with higher Curie temperatures at 1273 K can still be used.^29,30^

A pulse-receiver instrument sends a signal to the transducer. Transducer pulse generation details are commonly not fully described, but in 1967 Davis and Gordon stated their system had a 5 Mc/s pulse of 10 ls duration at 60cps repetition.^31^ When a transducer is used to generate sound waves a cylindrical buffer rod is commonly employed to either (a) isolate the temperature-sensitive transducer from the measured sample—in the case of PZT for high temperature methods, or (b) act as a wave guide—in the case of LiNbO_3_ transducer use in the large volume press (LVP) and diamond anvil cell (DAC).^32^ Unfortunately, for lower pressure methods the attachment method between the transducer and buffer rod is commonly not described but include: joining with glue, soldering, brazing, and dry contact or diffusion bonding.^33^ Attachment methods for the transducer to the buffer rod are recorded by Kleppa^34^ using phenyl salicylate, and Leibowitz *et al.* noted the use of epoxy cement Hysolresin (R8–2038) and Hysol Hardner (H2–3490).^35^ In LVP, a 2.5 *μ*m thick gold foil is commonly positioned on either end of a buffer rod (Al_2_O_3_) to maximize ultrasonic energy transmission. There is a lack of discussion or recognition in the literature if there are any adverse effects of this coupling layer with respect to sample purity.

A major consideration in designing the buffer rod is the diameter for suppressing trailing echoes. There are two sources of trailing echoes. First, from the emitted longitudinal pulse wave partly impinging on the sidewall, which is then reflected accompanied by harmonies. Second, converted shear waves can propagate along the rod causing reflection on the sidewall and convert into longitudinal waves but at a slower speed than the emitted longitudinal wave as shear waves are slower than longitudinal waves. This leads to spurious echoes from both the impinging sidewall and shear waves converting to longitudinal waves which are received. While smaller diameter buffer rods increase the design complexity to avoid the aforementioned issues, larger diameter rods are less desirable when considering thermal gradients, buffer rod material availability, and cost. One can refer to the work by Ai and Lange for examples of calculations that should be done to optimize the geometry of ultrasonic interferometric setups.^27^

In methods that operate at high pressures, like LVP or DAC, transducers (commonly 10° Y-Cut LiNbO_3_) with higher center frequencies (15–67 MHz) are used.^36–38^ The use of higher frequency acoustics for interferometric study of sound speed of materials in a DAC has advanced from MHz transducers to GHz, and most recently microwave resonator integrated directly onto a DAC.^39,40^ It should be noted that interferometry is hindered by the quasi-hydrostatic pressure effects that occur when the pressure transmitting medium (PTM) solidifies (typically above a few GPa).

#### 2. Laser

Laser sound generation involves the induction of a pulsed laser-generated stress wave to one side of a cylindrical wire sample surrounded by an inert gas, commonly argon or nitrogen. The stress wave spherically diverges and degrades into an acoustic wave that propagates through the sample and creates a disturbance in the surface motion of the surrounding inert gas. A streak recorder provides the transient time for the acoustic wave propagating through the sample of a known diameter, with corresponds to the emergence time of the wave. At ambient pressure sound generation into a sample via a laser is used in dynamic heating experiments, discussed in detail in Sec. IIC 2, beginning in 1986 by Hixson *et al.* and 1993 by Boivineau *et al.*^41,42^

At elevated pressures, like those in a DAC, three other methods have been used to measure condensed matter speed of sound at extreme conditions, outlined in Table I.

### B. Measurement uncertainty

Understanding measurement uncertainty is crucial for an experimentalist, database curator, and theoretical user. Two detailed references for uncertainty include: (1) the “Guide to the Expression of Uncertainty in Measurement” (GUM),^43,44^ which is a collaboration between the International Organization for Standardization (ISO) and the International Electrotechnical Commission (IEC) standard ISO/IEC 17025,^45^ and (2) additional international standard published by Joint Committee for Guides in Metrology (JCGM) JCGM100:2008.^46^ Pursuits in decreasing sound speed measurement uncertainty of pressure and temperature with standards were proposed by international committees like the International Practical Pressure Scale (IPPS) and the Consultative Committee for Thermometry’s Non-Contact Thermometry Working Group (CCT WG-NCTh).^47,48^

**FIG. 4.**
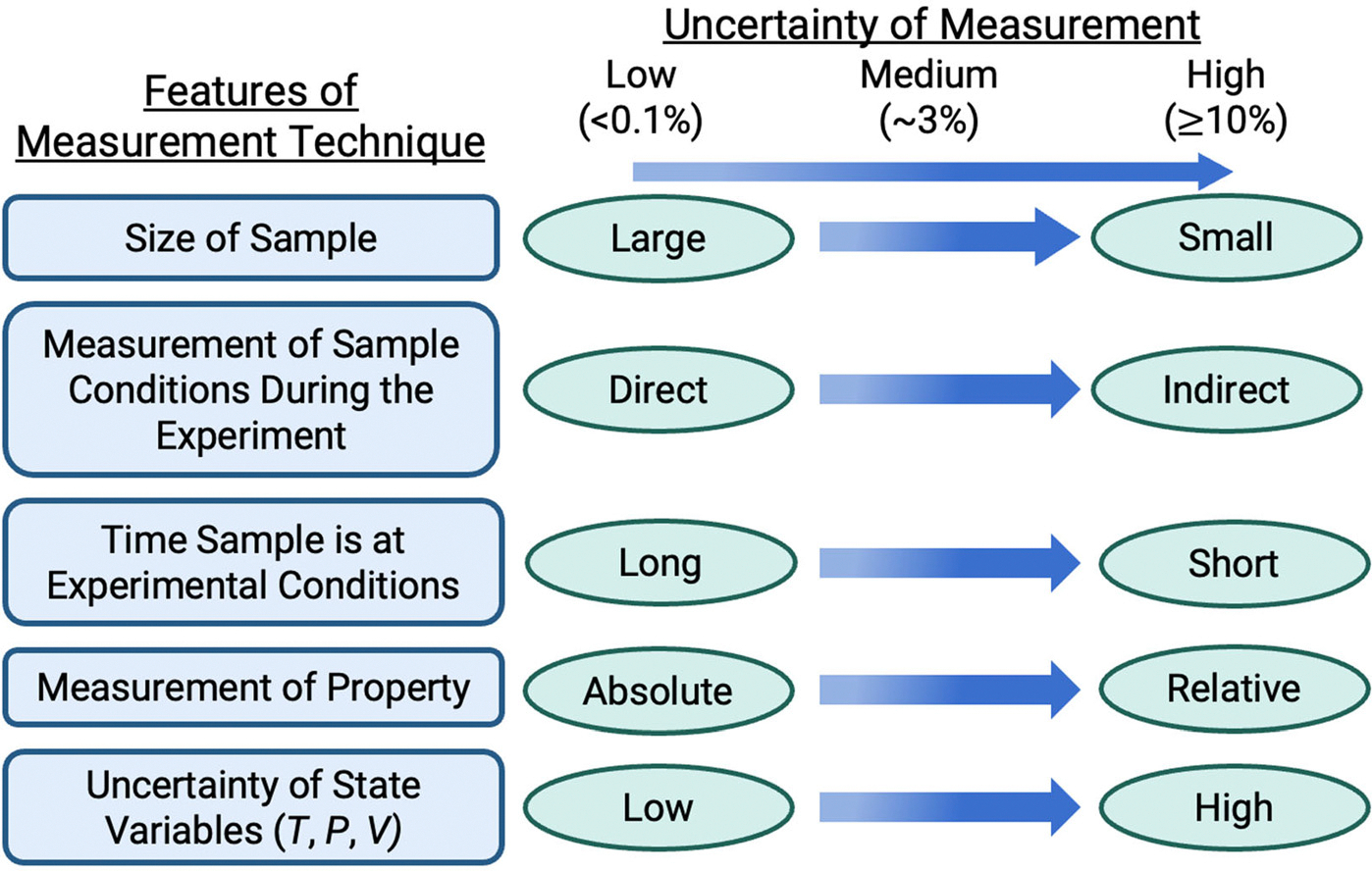
Schematic outline showing how features of measurement techniques affect the uncertainty of the resulting material characteristic measurements obtained.

In SoS literature, it has become common practice to report the sound speed uncertainty by taking into consideration uncertainty caused by geometric variables like the length of the sample (by a spacer or otherwise) or the uncertainty in the time variable like from electronic components like the oscilloscope, but this leaves out other important contributions. To report the overall SoS measurement uncertainty, components of the uncertainty must also include (a) uncertainties associated with impurities in and heterogeneity of the sample, and (b) systematic errors in the measurement process – in the case of speed of sound it is sample length and time, and (c) uncertainties in-state variables (*T, P, V*). An overview diagram relating these experimental setups and relationships to uncertainty is shown in Fig. 4. This section outlines some key considerations and effects for measurement uncertainty on apparatuses reported in the past 20 years and for future apparatus development.

#### 1. Sample purity

Any material contacting a sample should be well described, as impurities can impact the integrity of reported measurements. Researchers commonly choose not to note the purity of buffer rod and crucible materials, but when stated, purity is recorded as 99.98%–99.999%. Declaring material purity is especially important given the lack of post-measurement analysis of the sample purity reported in the literature. In SoS of metals at ambient pressure and up to 1500 K, Greenberg *et al.* stated that they chemically analyzed samples post-experiments to verify that no chemical reaction had occurred. Yet, the paper did not disclose details about what this chemical analysis entailed or the results of it.^49^ The ambient sample conditions during measurement need to be considered to assess chemical reactions of the sample during experiments. For methods below 280 MPa gas surrounding the sample commonly consists of “high-purity” (99%–99.99%) N_2_, Ar, Ar–CO mixture, Ar–H_2_ mixture, or He–H_2_ mixture. For higher pressure methods like LVP or DAC, the sample is in contact with the diamond cell and usually a standard optical pressure sensor material like ruby or Sm-doped strontium tetraborate (SrB_4_O_7_:Sm^2+^). A study analyzing the effects of the *in situ* pressure standards post-experiment to ensure no diffusion or reaction occurred has not yet been reported in the literature. The method that measures the speed of sound for condensed matter at extreme conditions that minimizes impurities is the container-less isobaric expansion experiment (IEX) technique performed over the years at Los Alamos National Laboratory and is discussed in Sec. II C2.^41,42,50^

#### 2. Temperature uncertainty

The effect on the overall temperature measurement uncertainty (*U*_*T*_) budget is commonly not reported but needs to be considered. By our accounts, over 80% of apparatuses in the literature published in the past 20 years use a type K thermocouple to measure the sample. Type K thermocouples are made of nickel–chromium vs nickel–aluminum with a measurement range of 73–1523 K.^51,52^ It should be noted that above 273 K type K thermocouples have a standard uncertainty of ±2.2 K or ±0.75%, whichever is greater, as defined by the American Society for Testing and Materials (ASTM) in ASTM E230–87:2017^53^ and two NIST Reports.^54,55^ With proper calibration uncertainties can be lowered significantly. An equivalent to ASTM E230–87:2017 standard limit of error is a Class 2 tolerance class defined by the International Electrotechnical Commission IEC-EN 60584.^56^ To put the uncertainty into perspective, at 1000 K a type-K thermocouple will report temperatures within a standard uncertainty of ±7.5 K. Selecting different thermocouples to record the temperature at different temperature ranges could decrease the uncertainty associated with temperature, but these thoughtful steps are commonly not carried out due to the added experimental complications and cost. For thermocouple selection, the size, calibration, and exposure of the temperature measurement device to extreme conditions should be considered, documented, and accounted for in measuring and reporting. If the thermocouple is at high pressure, the effect of pressure on the electromagnetic force of the thermocouple must also be accounted for.^57,58^ At higher temperatures, thicker wires should be used, but commonly the thermocouple wire diameter is not reported in apparatus papers. Although thermocouple drift is well documented, and temperature measurement devices should be calibrated on a schedule considering the length of time they are exposed to extreme conditions, this information is rarely reported.^52^ Calibration can be performed with high-temperature fixed points according to the international temperature scale from 1990 (ITS-90), which are defined up to copper’s melting-freezing point temperature of 1357.77 K at a pressure of 101.325 Pa. Carbon eutectics of Re–C, Pt–C, and Co–C at 2747.84 K ± 0.35 K, 2011.43 K ± 0.18 K, and 1597.39 K ± 0.13 K, respectively, are available to extend calibrations to an even higher temperature range.^59–61^ A 2023 review by Fellmuth and Gaiser is a good reference providing detail on high-accuracy realization for temperature fixed and reference points.^62^ Hartsfield *et al.* recently conducted shock compression experiments where Raman spectroscopy and optical pyrometry were used as dynamic temperature measurement methods, concluding that the results are consistent with each other although the uncertainties were larger than other methods and they concluded that further work was needed to settle on a best-practice temperature measurement in the shock compression technique.^63^ Issues with diffraction temperature measurements were also noted by Sharma and Gupta for x-ray diffraction (XRD) methods.^64^ In 2022, Dornheim *et al.* proposed a method to extract temperature from x-ray Thomson-scattering experiments with special emphasis on accuracy and free from approximations that could be useful in facilities like the LCLS, European XFEL, or SACLA.^65^ The reader is referred to a recent review by Hartsfield and Dolan on specifics for temperature measurement in dynamic compression experiments.^66^

#### 3. Pressure uncertainty

In addition to the contribution of temperature measurement uncertainty in experimental measurements, pressure measurement uncertainty (*U*_*P*_) is commonly under-reported in the literature. To begin with, most papers do not report the ambient pressure conditions, which although the effect is assumably small, represents poor reporting practice. For pressures created in setups using a pressurizing fluid up to 280 MPa, experimental pressure can be measured using commercially available pressure transducers that employ resonant quartz crystals where the frequency of oscillation varies with pressure-induced stress.^67^ The highest quality pressure transducers can have parts-per-billion resolution and accuracy up to 0.01% of full scale. When experiments use a pressure transducer for measurements up to 280 MPa the calibration efforts to ensure the accuracy of readings should be discussed. Calibration for pressure transducers using a gas or oil working fluid allows pressure calibration up to 104 and 280 MPa, respectively.^68–70^

For speed of sound experiments operating above 280 MPa like in diamond anvil cell or anvil press, experimental pressure is measured by using pressure standard reference materials such as MgO by Zha *et al.* and more recently, Ruby by Shen *et al.*^47,71^ The optical pressure sensor utilizing the Ruby pressure standard reference material, Sm-doped strontium tetraborate (SrB_4_O_7_:Sm^2+^), reports uncertainties with a range of ±0.2 GPa.^72^ Additionally ruby as well as a mixture of Au, Pt, and NaCl powder can be added to the sample chamber for pressure estimation and cross calibration. A working group of the International Practical Pressure Scale (IPPS) has endorsed a ruby pressure gauge, “Ruby2020”.^47^ These optical pressure sensor methods are well established and considered best practice while still having rather large uncertainties with reported pressures under 2 GPa having relative deviations of over 10%. This can impact the material’s defined critical points and melting curves.^73^ Researchers at the U.S. Department of Energy national laboratories, namely, Sandia and Lawrence Livermore National Laboratory, have recently also investigated pressure standard up to 1 TPa using Au and Pt for shock compression experiments.^74^ More discussion on pressure and uncertainty for experiments over 1 GPa is included in Secs. II D2 and II D3.

#### 4. Uncertainty in homogeneous conditions

Thermal and pressure gradients across a sample must be considered, as they will occur with sample sizes of all ranges. Thus, the required measurement time of a measurement technique is important to consider. If the sample is at experimental conditions for a longer time, then it is more likely that the bulk material is at homogeneous conditions, and the measurement result represents constant state variable conditions. Steady-state and quasi-steady-state techniques hold the specimen at conditions from a few seconds to hours. Examples of techniques that hold a specimen at steady-state temperature are ultrasonic interferometric techniques discussed in Sec. II C1. Greenburg *et al.* disclosed that at each measuring point, the furnace was held at a constant temperature for 10 min to attain thermal equilibrium before starting a measurement.^75^ In the reviewed literature temperature conditions were noted to be held between 10 and 30 min, but it is unclear if thermal equilibrium was actually met given the sample volume of 1 cm^3^ (in the case of Greenburg *et al.*) or larger. It is admirable that Greenburg *et al.* did note a total of 10 repeated measurements at each temperature, which adds confidence of equilibrium, if in fact 10 min between each measurement was given. A good example for an apparatus where steady-state conditions are rigorously pursued is in a publication by Outcalt and McLinden where they described that measurements of the temperature and pressure being monitored every 30 s with equilibrium being defined as when over 15 consecutive minutes, the standard deviation in the temperature was ≤3.5 mK and the standard deviation in the pressure was ≤5 kPa; only after these convergence criteria were met, data were recorded and averaged for 5 more minutes.^76^ The advantage of experiments completed in time scales on the order of millisecond to sub-microsecond resolution is that more extreme conditions can be measured. For example, with dynamic heating techniques, discussed in Sec. IIC2, temperatures above 10 000 K can be reached, and the shock compression technique discussed in Sec. IID enables measurements up to 600 GPa. Thus, a trade-off is made between the experiment’s operation range and the uncertainty of the sample’s measurement conditions. Discussion on the trade-off of a method’s goal and uncertainty is presented in Sec. IIE.

In parallel to ensuring equilibrium with the surrounding the temperature gradients can be reduced with proper insulation, decreasing convection in furnaces, positioning a copper sleeve around the sample area, or implementing a heat pipe lining to the furnace. Heat pipes with materials like sodium or silver can operate from 370 to 2500 K and 455 to 3500 K, respectively, with gradients of ±80 mK. Custom pressure-controlled heat pipes have been designed and tested by national metrology laboratories, like the United Kingdom’s National Physical Laboratory, such that thermal gradients of ±5 mK have been reported.^77^ Thermal gradients need to be considered even in the smallest samples, like in diamond anvil cells. For reducing pressure gradients in a diamond anvil cell, one should ensure the sample is centered in the cell. Attaching directly to an anvil requires soft materials; still, ~0.5 GPa gradient was observed over a 2 mm thick sample.

### C. High-temperature methods

This section presents high-temperature speed of sound techniques, herein defined as instrumentation that conducts experiments above 600 K and up to 1 MPa. Methods that perform experiments above 1 MPa and at high temperatures are discussed in Sec. II D. First, Sec. II C 1 covers steady-state heating techniques that are limited to under 2500 K. Sound speed is measured in these systems using ultrasonic interferometric methods. Section II C2 covers dynamic resistive heating and dynamic laser heating techniques where the speed of sound is generated by measurements below 1 MPa and at temperatures over 10 000 K.

It should be noted that the electromagnetic levitation technique uses the dynamic resistive heating technique to measure thermophysical properties of condensed matter in magnetic materials at temperatures up to 3000 K, but the technique measures density and surface tension, not speed of sound.^78^ Thus, electromagnetic levitation technique will not be covered here as it is not a method for speed of sound measurements. A review by Brillo *et al.* provides additional information on levitation techniques.^79^

#### 1. Resistive heating: 600–2500 K

**FIG. 5.**
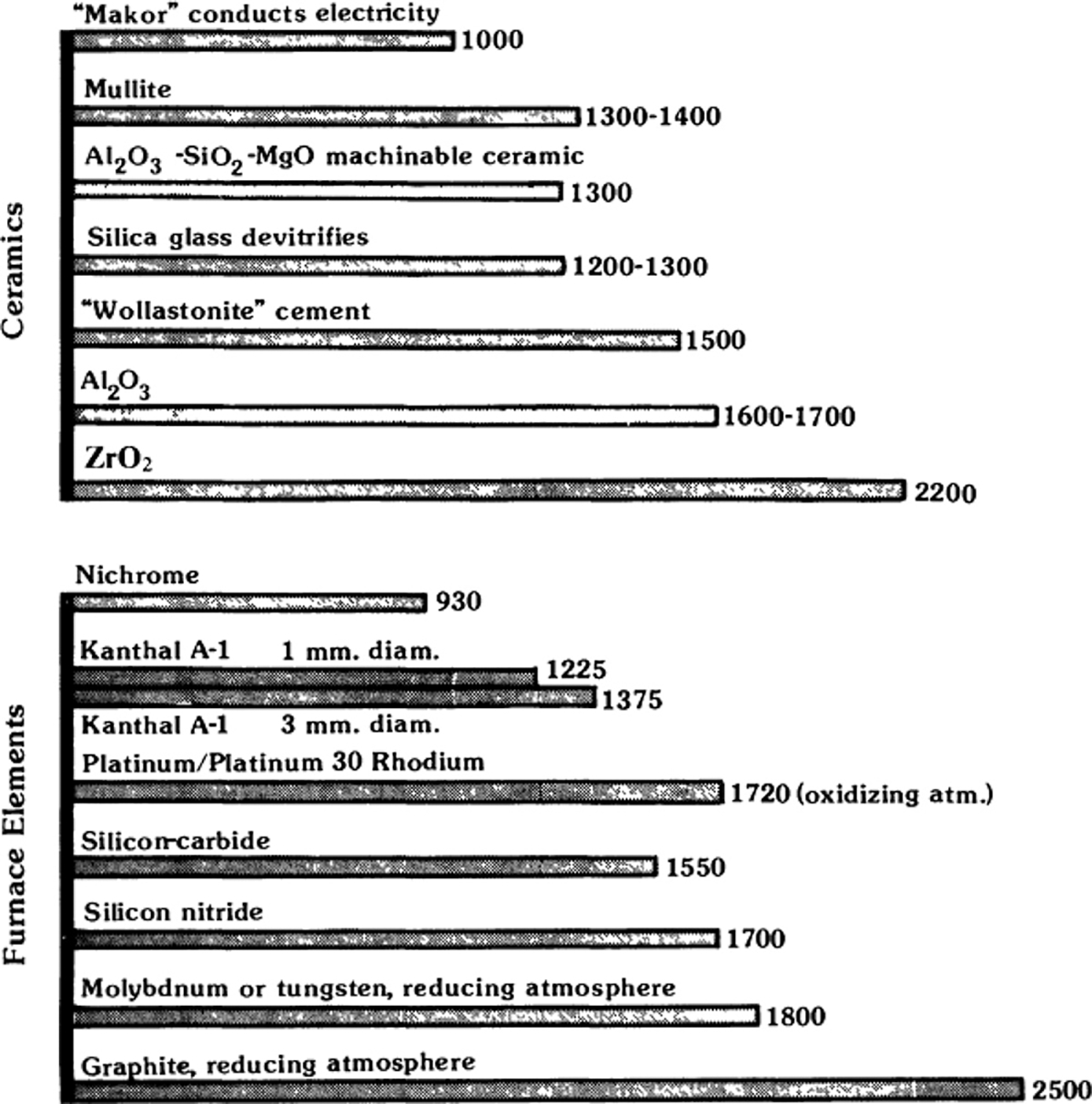
Temperature ranges (°C) for some ceramic and furnace element materials. Image from Holloway and Wood,^81^ Reproduced with permission from Springer Nature (2012). Copyright 1988, Springer Netherlands.

Resistive heating methods like an internal or external furnace can enable measuring the speed of sound up to 2500 K. The furnace element type selected, Fig. 5, will affect the temperature range obtainable; for some materials a reducing atmosphere will also be needed. An induction furnace method to characterize molten metals was recently published by Dejaeghere *et al.* allowing for experiments at temperatures above those available with resistive heating elements, up to 2800 K.^80^

Resistive heating is commonly associated with the historic steady-state ultrasonic interferometric technique to measure a material’s speed of sound. Ultrasonic interferometric techniques can be divided into two classes: pulse-echo or pulse-transmission. The pulse-echo method was first reported by Pellam and Galt in 1946 as the “electronic pulse-circuit technique” and has since remained the basis for sound speed measurements in condensed matter below 2500 K.^82^ In 1949, Kleppa expanded the temperature range of the pulse-echo method to measure the liquid phase sound speed of 12 pure elements (Zn, Ga, Cd, In, Sn, Tl, Pb, Bi, Na, K, Rb, Cs) and 4 equi-atomic mixtures (Sn–Cd, Sn–Hg, Sn–Bi, Sn–Pb).^34^ The present manuscript focuses on recent condensed matter speed of sound developments, so further historical discussion of ultrasonic interferometric techniques is not included. As Sec. II C focuses only on high-temperature methods below 1MPa, discussion on ultrasonic interferometric technique to measure the speed of sound for resistively heated samples at high-pressure (via pressure vessel, anvil press, and DAC) is reserved for Sec. II D.

**FIG. 6.**
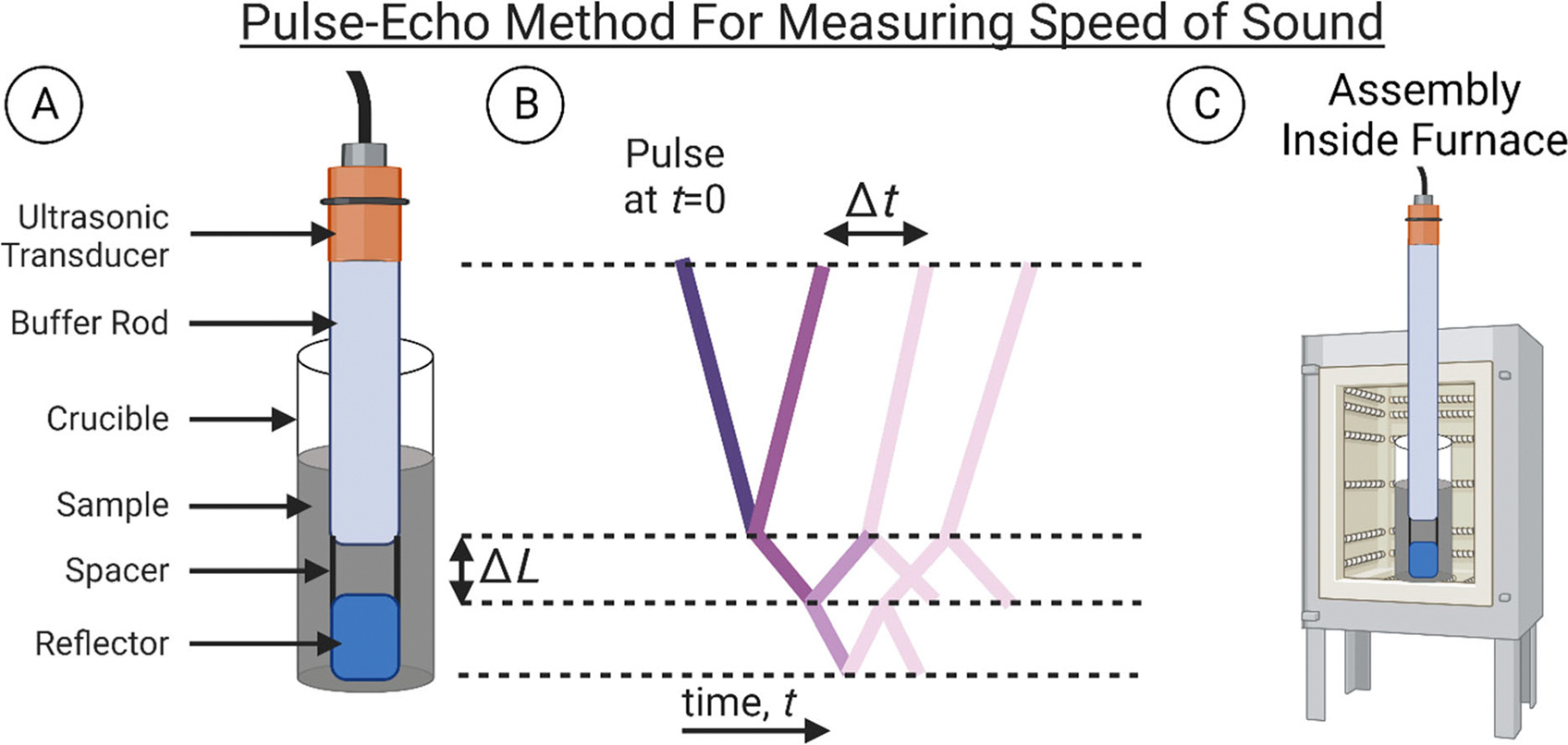
Schematic of the pulse-echo method to measure the liquid phase speed of sound: (a) instrument components, ΔL indicates sample length; (b) diagram of the pulse with Δt indicating the arrival time of echoes and lighter colors indicating lower signal strength; (c) orientation of assembly inside the furnace for high-temperature experiments.

Figure 6 is a schematic diagram of the pulse-echo method to measure the speed of sound in a liquid phase material. The basic principle of the pulse-echo method consists of a MHz ultrasonic transducer transmitting a pulse through a buffer rod (Al_2_O_3_ ceramic or Mo of lengths: 16–40cm; diameter = 1.5–3 cm) into a sample filled crucible (commonly of Pt, Mo, and Al_2_O_3_) and then an oscilloscope receives and records a first echo from the interface of the buffer rod and the sample and a second echo from the interface of the sample and a reflector. For a liquid phase sample, a spacer can be used to define the sample length, L, from the end of the buffer rod to the reflector. The speed of sound, c, is then calculated from the time, Δt, for echoes to travel the length of the buffer rod and the length of the sample, $c\;=\;\frac{2\Delta L}{\Delta t}$. In some pulse-echo set-ups a reflector is used, and reflectors are commonly the same material as the crucible, but alternatives of WC have been used.

Since 2000, variations to the pulse-echo method have included a frequency sweep acoustic interferometer apparatus by Ai and Lange of the University of Michigan in 2004.^27^ The apparatus by Ai and Lange can measure liquid phase samples at atmospheric pressure and temperatures up to 1975 K using a transducer with a center frequency transmission range between 3 and 7 MHz to analyze if the sound speed measured is frequency-independent indicating a relaxed sample material. Lange’s group has used the frequency sweep acoustic interferometer apparatus to report the speed of sound of silicate^27^ and carbonate melts.^83^ A shortcoming in the acoustic interferometer apparatus reporting is the lack of uncertainty analysis associated with temperature measurement that could stem from the (a) temperature measurement device, and (b) thermal gradients in the sample. A type K thermocouple was used which has ±2.2% uncertainty, and no calibration of the thermocouple was disclosed. Additionally, given that the large sample size (~9 cm^3^ given the disclosed crucible size of 2.4 cm diameter and 2cm depth) in the apparatus, temperature gradients could exist within the sample. Thus, the temperature uncertainty could add a substantial amount of up to ±1% expanded combined uncertainty. The shortcomings in the method report are not unique to this research group, but rather indicative of almost all reviewed reports having similar lack of details in uncertainty analysis—especially with respect to temperature.

**FIG. 7.**
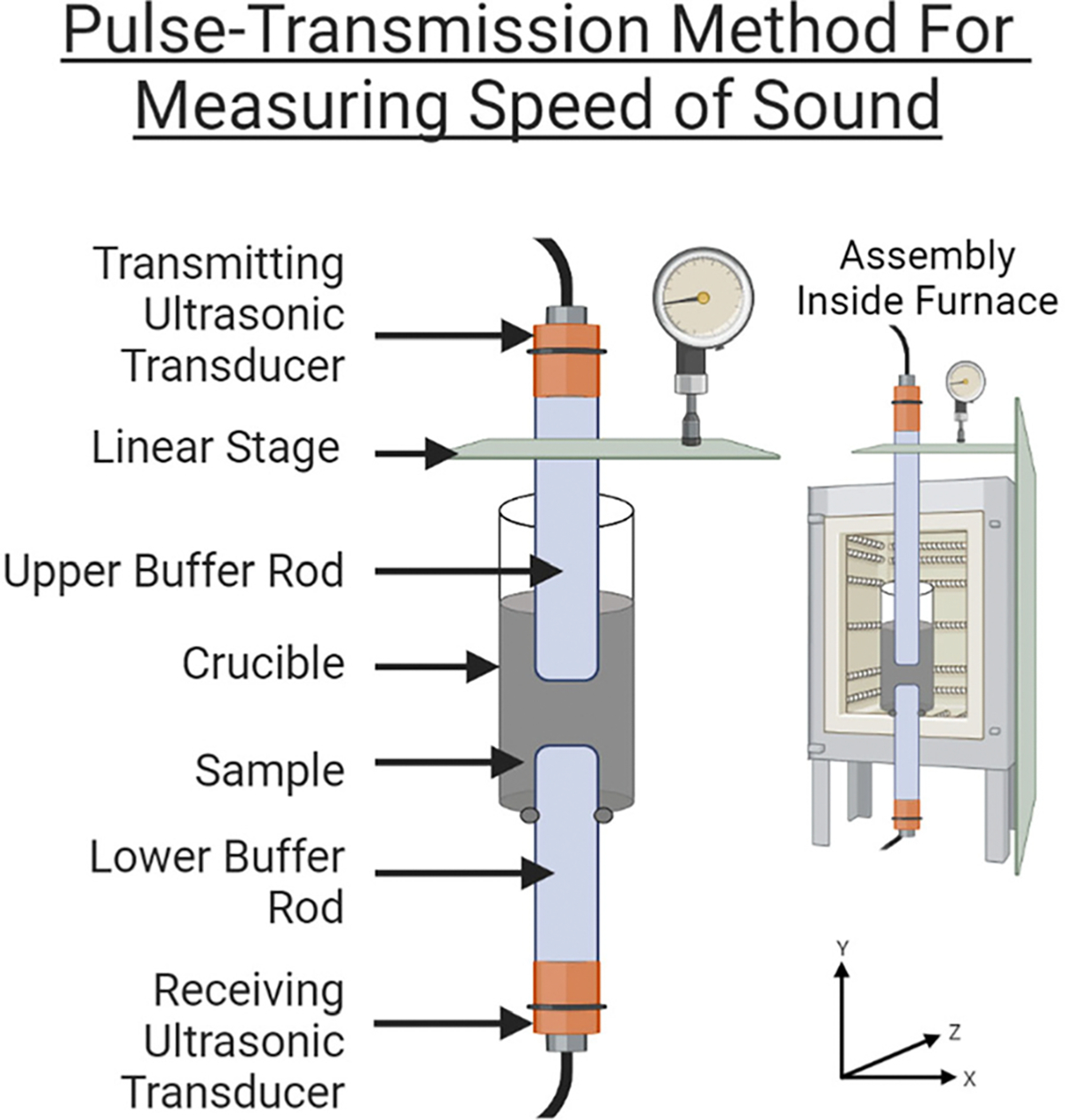
Schematic of pulse-transmission method to measure the speed of sound for samples in the liquid phase. A “transmitting” ultrasonic transducer sends a pulse through an upper buffer rod to a sample contained in a crucible and a lower buffer rod passes the echoes to the receiving ultrasonic transducer. At a chosen experimental condition, the length of the sample is adjusted to different positions defined by moving the upper buffer rod along the y axis by a micrometer.

A variation of the pulse-echo method is the pulse-transmission method (Fig. 7). The pulse-transmission method substitutes the reflector in the pulse-echo technique for a second buffer rod with a second ultrasonic transducer attached to the end, which receives the pulse sent by the first ultrasonic transducer. Instead of employing a spacer to define a fixed consistent sample length, this technique uses a “varying sample length”. The sample length is varied by moving the buffer rod vertically within the liquid sample. The movement of the buffer rod is measured by a micrometer.^75^ Either transducer can be used as transmitter or receiver.

A pulse-transmission apparatus reported in 2007 by Hayashi *et al.* of the Tokyo Institute of Technology^84^ measured liquid phase Pb, Sn, Ge, and Si from *T*_m_ to 1880 K at atmospheric pressure. An atmosphere of Ar and He was employed to reduce corrosion, similar to the 1982 pulse-transmission apparatus by Tsu *et al.*^85^ who measured liquid phase Bi, Al, Au, and Cu. In 2008, Greenberg *et al.* of Ben Gurion University of the Negev, with collaboration with other researchers from across Israel, reported a custom pulse-transmission apparatus to measure the liquid phase sound velocity of Pb, Sn, Bi, and Sb from *T*_m_ to 1408 K at atmospheric pressure.^75^ Due to inaccuracy in path length measurements, the Greenberg *et al.* group later modified their apparatus to be a variable-path length pulse-echo method with a reduced maximum operating temperature of 1063 K for measurements of Bi–Sb alloys,^86^ Ga, In,^87^ and recently six alloys of In–Ga,^88^ with all experiments performed in an Ar environment at atmospheric pressure. Unfortunately, the research group has yet to disclose many basic design parameters of the apparatus including the diameter of the buffer rods, attachment methods between the transducer and buffer rod, or ambient pressure measurement, which would serve to validate uncertainty claims.

#### 2. Laser heating and resistive self-heating: 2500–10 000+ K

In steady-state techniques the long duration (1 s to many hours) of the specimen’s exposure to high temperatures leads to experimental difficulties, especially above 2500 K. These experimental difficulties include chemical reactions, sample contamination, selective evaporation, loss of mechanical strength, heat transfer, and breakdown of insulation.^26^ Nevertheless, measuring sound speed above 2500 K is desirable for many reasons including defining the critical points of materials with high melting points, such as refractory metals. Dynamic heating methods allow for the measurement of thermophysical properties of condensed matter materials to over 10 000 K. Two dynamic heating methods to perform experiments above 2500 K are (1) laser heating and (2) dynamic resistive self-heating.

##### a. Laser heating.

Laser heating is a technique that utilizes a focused laser beam to raise the temperature of a material. Dynamic laser surface heating is employed in many experimental setups to study materials under extreme conditions and involves using a continuous wave (CW) or ns pulsed laser source to heat a sample. Heating the sample is assumed to be a coupled two-step process of the laser-metal interactions where a thin layer of the material absorbs photon energy by electrons, and then the metal lattice is subsequently heated via electron-phonon collisions. This two-step process leads to the method being considered as surface heating. A CW laser has the advantage of being available at high powers, while a pulsed laser can have extreme energy density and a wide range of pulse durations.^26^

Having a non-contact method with precise, rapid, and uniform control over the sample heating has been a key advancement to study the melting phase relationships of U and Pu–O systems. For example, in 2014 when Böhler *et al.* studied nuclear-rich materials they found that historically published methods using a crucible to hold the sample led to an oxygen mixture effect that caused inaccuracies in the solid to liquid phase diagram.^89^ The same group also looked at the solidification of UO_2_–ThO_2_ systems with laser heating.^89^ Higher temperatures of up to over 4000 K have been achieved by Cedillos-Barraza *et al.* in 2016 for TaC–HfC systems used in hypersonic vehicles.^90^ Laser heating also has a very high energy density, which is particularly adventitious in applications requiring localized heating or materials with high thermal conductivity, like the elements in scope for this review.

Laser heating is advantageous as it can be seamlessly integrated with various *in situ* characterization techniques such as spectroscopy, microscopy, and diffraction. This enables real-time monitoring and analysis of the material’s response to temperature changes, offering valuable insights into phase transitions, structural transformations, and other dynamic processes. A review by Elhadj *et al.* describes infrared thermal imaging and laser heating for studying thermophysical properties at high temperatures.^91^ A review on laser heating by Manara *et al.* goes into more detail.^92^

##### b. Resistive self-heating.

**FIG. 8.**
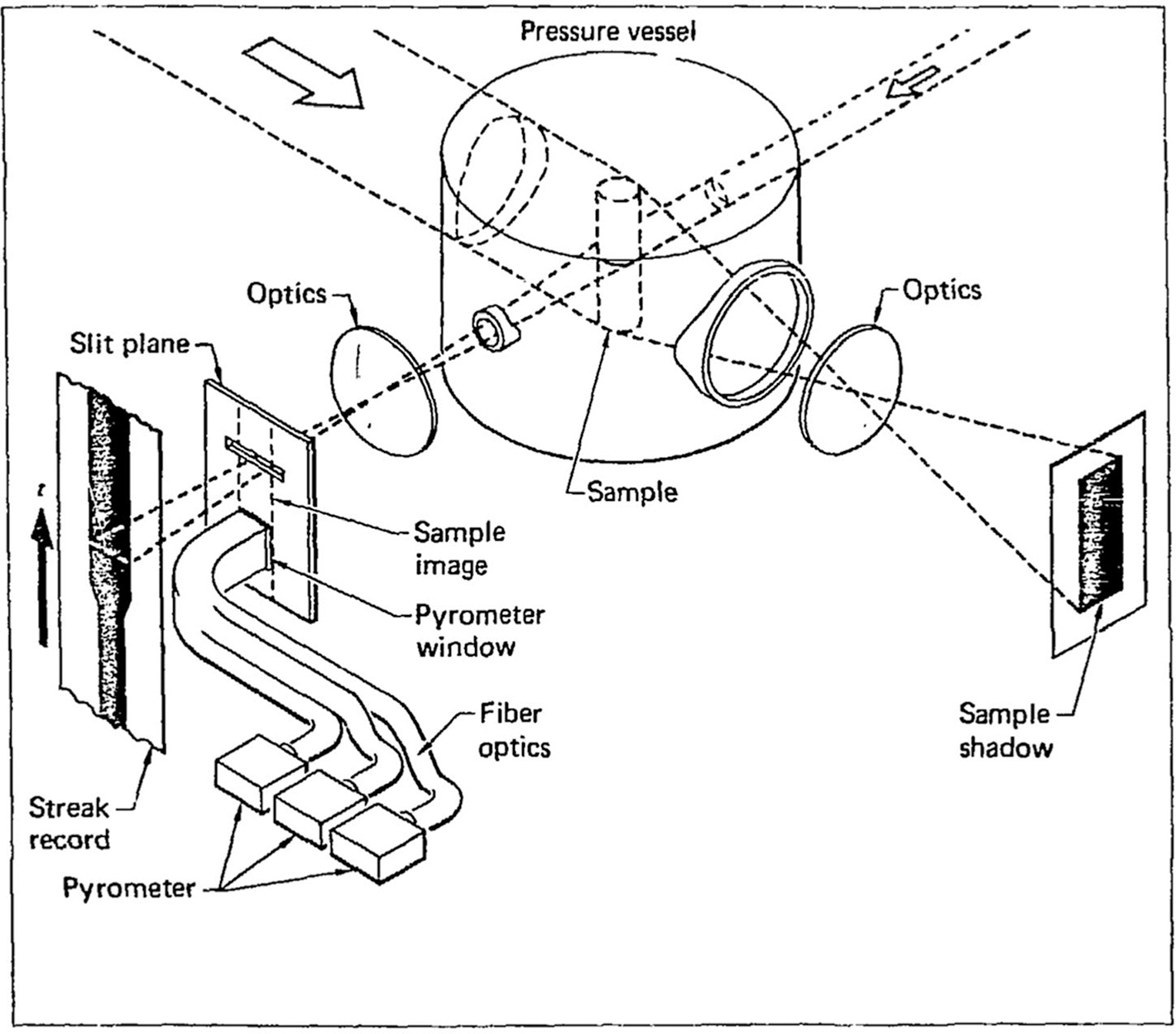
Schematic representation of the principal diagnostics used with the isobaric expansion experiment. Backlighting with lasers in two orthogonal directions casts shadows of the sample. In one line of sight, the entire shadow is recorded for symmetry information. In the other, a slit is used to define a particular sample diameter and a streak record is made to determine dynamics. An optical pyrometer views the sample in the shadow region. Reproduced from Hodgson Dissertation, 1978; licensed under a Creative Commons Attribution (CC BY) license.^101^

For speed of sound measurement methods reaching temperatures above 3000 K, dynamic resistive (Ohmic) self-heating techniques have also been used. In self-heating techniques, also known as “explosion of metal wires” or “wire explosion”, a Joule effect is created by applying an electrical current over a short time period (<1 ms) to a sample in a wire (typically 1 mm diameter, 25 mm length) or strip geometry. The wire sample is resistively heated in an inert gas atmosphere by a square voltage pulse at a high current (15 × 10^3^ A). The wire sample is backlit with an argon CW laser and then a ruby pulsed laser creates a stress wave in the sample and speed of sound is calculated. In the experiment, enthalpy, temperature, and specific volume are measured as functions of time, and sound velocity is measured at the end state. One of the limitations of this method is the higher experimental uncertainty between ± 5%–7%, with large contributions from the pyrometric temperature measurement, described in Sec. II B2. One of the benefits is that it is a containerless method, so sample contamination and chemical reactions are minimized. Research with this technique started in the late 1970s at Lawrence Livermore Laboratory by Gathers *et al.*^93^ This work was continued in 1986 when Hixson and Winkler studied a resistive pulse-heating method to investigate liquid-metals at high temperatures and constant pressures up to 1 MPa.^41^ The technique was named the isobaric expansion experiment (IEX) and has been used to study the melting point of many elements mainly from 1985 to 2001, and is the basis for many solid and liquid phase standards in the ITS-90.^94^ In the past five years, the data created in the 1970–2005 time period has been used to guide many theoretical and computational studies.^95,96^ Figure 8 is a schematic of IEX by Hixson *et al.*^97^

As dynamic resistive self-heating techniques execute in such short timescales and at such high temperatures, temperature measurements must be performed by radiation thermometry, also known as optical pyrometry. Pyrometers measure the surface temperature by analyzing the light emission from the heated sample. Advancements in pyrometry include multi-wavelength pyrometry and polarization pyrometry. For pyrometric temperature measurement techniques the lack of accurate emissivity data of materials is a major source of uncertainty.^98^ Further details on surface temperature measurements and pyrometry are available in recent reviews by Zhang and Araújo. ^99,100^

The reader is directed to a review by Boivineau and Pottlacher for additional details on dynamic heating methods to obtain thermophysical properties beyond the speed of sound.^26^ A 2020 review by Oreshkin and Baksht provides a review of explosion of wires in vacuum with a special focus of extreme conditions ranging from condensed matter phases of solid and liquid to plasma.^102^

### D. High-pressure methods

High-pressure techniques are defined as instrumentation that can conduct experiments at or above 0.1 GPa. Presented in order of increasing pressure range, the first discussed method to reach high pressures is using a pressure vessel (common: 0.1–0.2 GPa; max: 1.0 GPa) constructed of a superalloy (Sec. II D1). Then, in Sec. IID 2, the anvil press (common: 1–30 GPa; max: 130 GPa), and notably the Paris–Edinburgh press, will be presented, followed by diamond anvil cell methods (common: 1–150 GPa; max: 300 GPa). Within a DAC, sound speed has been measured using ultrasonic interferometric methods, Brillouin scattering, inelastic x-ray scattering (IXS), and most recently via picosecond acoustics. The final high-pressure method to measure the sound speed of condensed matter materials (Sec. II D3) is the shock compression method (common: 100–300 GPa; max: 600 GPa). The scope of discussion is focused on experimental measurements completed in the past 20 years. However, where appropriate a reference toward original techniques is included to aid historical context for the reader.

#### 1. Pressure vessel: ≤ 1 GPa

**FIG. 9.**
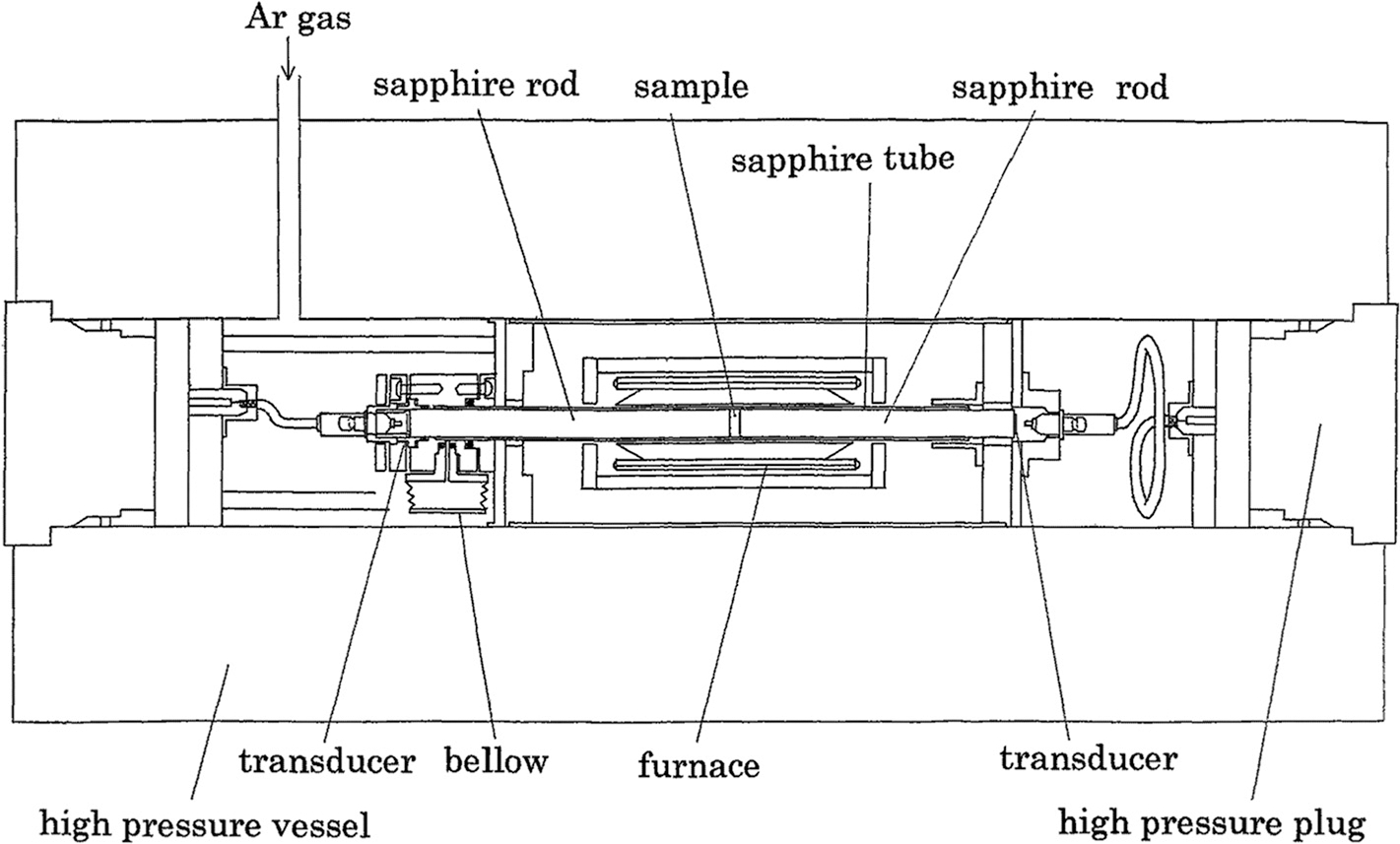
Schematic of the experimental apparatus to measure liquid phase speed of sound for metals up to 1843 K and 198 MPa.^103^ The apparatus continued to be used to study selenium^104^ and mercury.^105^ Image from Kohno and Yao.^103^ Reproduced with permission from J. Phys.: Condens. Matter **11**(28), 5399–5413 (1999). Copyright 1989, IOP Publishing Ltd.

In the past 20years, two groups have used an ultrasonic interferometric technique inside a pressure vessel to conduct solid and liquid phase speed-of-sound experiments up to 200 MPa and 1843 K. First, in 1999 Kohno and Yao at Kyoto University reported an internally heated high-pressure-vessel pulse-transmission apparatus (Fig. 9) to measure liquid Hg speed of sound from 120 to 198 MPa and 1429 to 1850 K.^103^ In the Kohno and Yao apparatus, argon gas pressurizes a bellows filled with the sample that pump it into a defined space (between 0.3 and 7 mm) between two buffer rods. The instrument can operate up to 198 MPa and 1843 K with temperature measured by two ITS-90 calibrated type-C thermocouples (WRe5%–WRe26%), and pressure measured by a Heise gauge. Uncertainty in temperature and pressure was stated as ±3 K and ±0.5 MPa, respectively. Yao continued using this apparatus and after modifications that decreased the temperature uncertainty to ±1 K contributed measurements on liquid phase Se^104^ and extended upon their initial study of liquid phase Hg at conditions of 1025–1975 K and 108–186 MPa.^105^ In the past 15years, no further investigations have been reported using this apparatus.

**FIG. 10.**
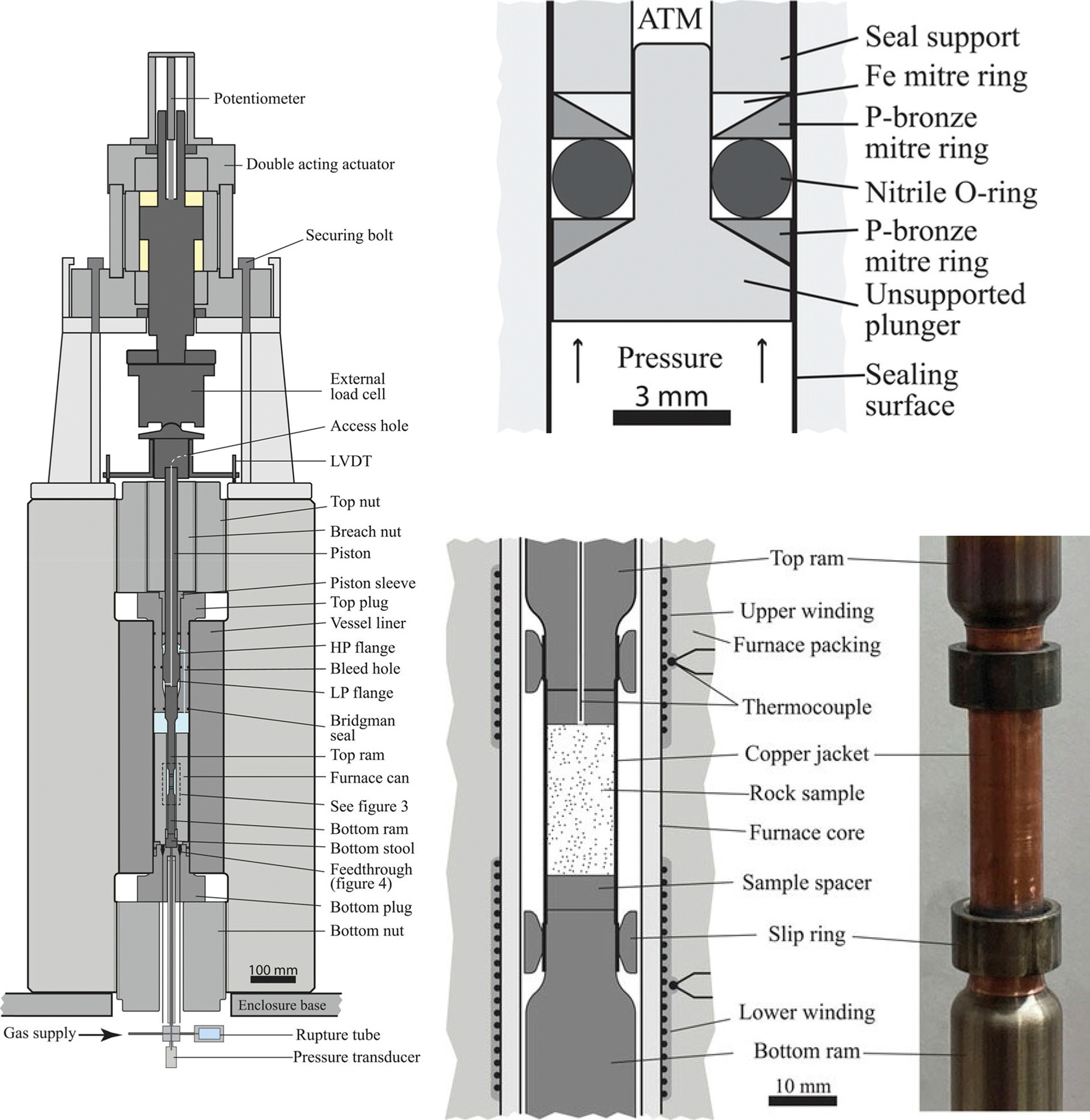
Annotated scale sectional view of the Murrell deformation apparatus. Apparatus can measure the speed of sound for solid phase materials up to 1273 K and 1000 MPa. Image from Harbord *et al*.^106^ Reproduced from Harbord *et al*., Rev. Sci. Instrum. **93**(5), 053908 (2022), with permission from AIP Publishing.

In 2022, Harbord *et al.* of the University College London modified a high-pressure vessel-based apparatus named the “Murrell” (Fig. 10), which was used to measure solid phase limestone samples from 433 to 673 K and 1 to 200 MPa.^106^ Harbord *et al.* state that the full range of the instrument is up to 1000 MPa (1 GPa), and with minor modifications, could conduct experiments at temperatures of 1273 K (1000 °C). Two type-K thermocouples measured the furnace temperature, and one type-K thermocouple measured the vessel temperature. No discussion on pressure measurement method other than the use of a gas booster was disclosed, and no uncertainty in pressure, temperature, or state variables was mentioned. The basics of the Harbord *et al.* instrument are from the 1970 work of Patterson, who measured rock deformation at temperatures up to 1773 K and pressures up to 300 MPa (at which time instrument failure occurred for experiments conducted at 500 MPa).^107^

In summary, the technique of utilizing a pressure vessel to gauge the speed of sound has been effectively employed for both solid and liquid phase materials. This range encompasses pressures from atmospheric—1 GPa and temperatures spanning 300–1700 K. As of the composition of this paper, there is a noticeable uptick in solid phase studies. These are predominantly centered on multicomponent materials, many of which have direct relevance to geological samples. These studies often explore the intricate structures and behaviors of composite materials, aiming to unlock a deeper understanding of Earth’s subsurface processes. On the other hand, investigations into the speed of sound in the liquid phase within a pressure vessel have predominantly focused on pure elements. The choice of such elements is not arbitrary; they hold significant potential for a plethora of industries. This includes the energy sector, where understanding the behavior of liquids at extreme conditions has applications in fusion and geothermal energy. The defense sector also stands to gain with further first principal analysis, especially in areas that require insights into the behavior of liquids under extreme conditions, which could be crucial for the development of next-generation equipment. Finally, the manufacturing industry can benefit from this knowledge, especially in processes that involve high-pressure applications, ensuring more robust and efficient production lines in fields such as EUV lithography in semiconductor manufacturing.

#### 2. Anvil press and diamond anvil cell: 1–300 GPa

**FIG. 11.**
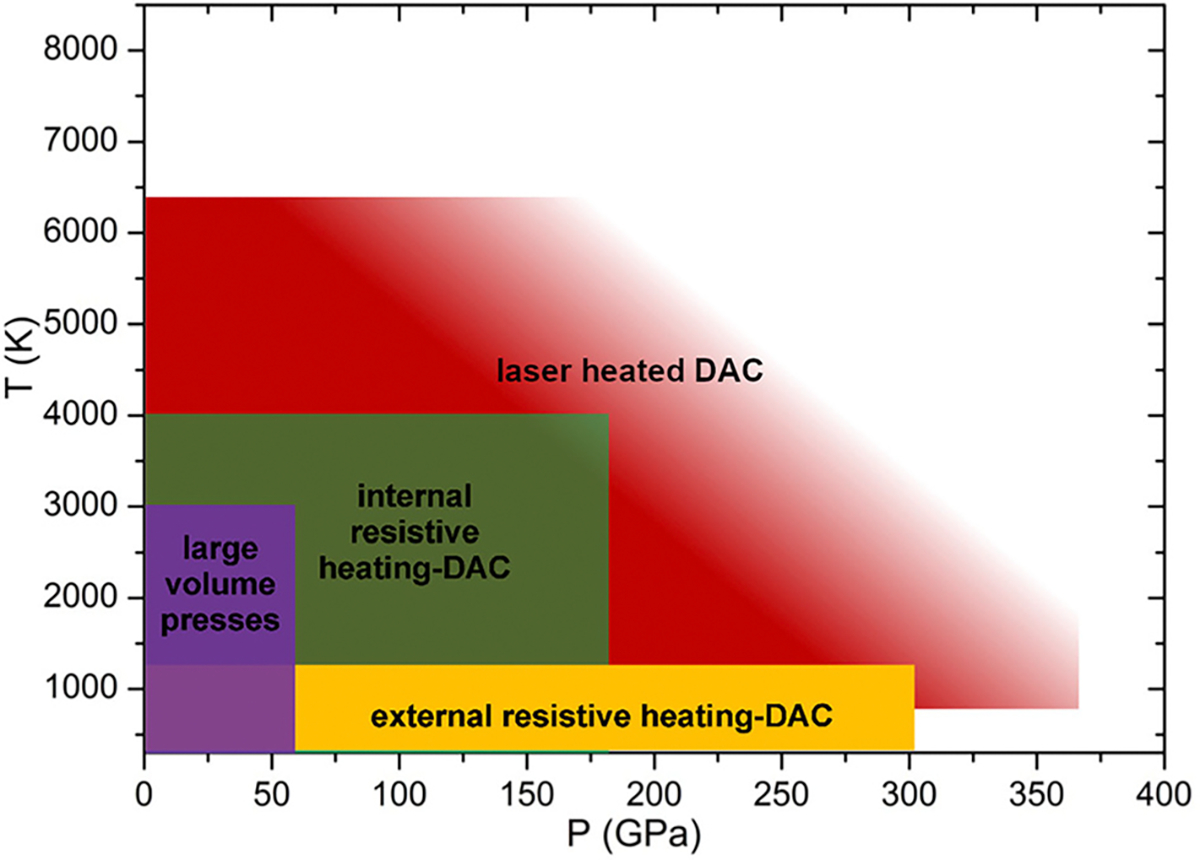
Comparison between the different *P* and *T* domains reachable using large volume press (LVP) and diamond anvil cell (DAC) static techniques.^110^ Anzellini and Boccato, Crystals **10**(6), 459 (2020); licensed under a Creative Commons Attribution (CC BY) license.

To measure the speed of sound for condensed phase materials at static pressures above 1 GPa two options are established: (1) an anvil press, also known as a large volume press (LVP), or (2) a diamond anvil cell (DAC), which can be used alone, or combined with a LVP. The first speed of sound measurements for condensed phase materials in a LVP or DAC were realized using ultrasonic interferometric methods by McSkimin in 1950^108^ and by Spetzler *et al.*^109^ in 1992. A 2014 review by Li and Liebermann goes into detail on interferometric methods for condensed matter materials at extreme conditions. Research in the past 20 years has worked to increase the (a) pressure, (b) sample size, (c) temperature, and (d) simultaneous *in situ* measurements of these two static high-pressure techniques. Increasing experimental pressure limits to 300 GPa has come with the transition from anvils made of tungsten carbide (WC) in LVP to DAC in an LVP. Increasing the sample size has been achieved by changing the geometry of the anvils. Advancement from ambient temperature conditions to elevated temperature up to 6000 K occurred with application of external resistive heating, to internal resistive heating, to laser heating, Fig. 11.

The ability to perform multiple *in situ* measurements simultaneously has progressed so that the speed of sound/acoustic properties (via ultrasonic interferometric or spectroscopic techniques), crystallinity properties (via x-ray diffraction), vibrational and symmetry properties (Raman scattering, infrared spectroscopy), plus other information can be extracted in one experiment. It should be noted that as more extreme conditions are reached the uncertainty of the measurement increases dramatically.

##### a. Anvil press.

**FIG. 12.**
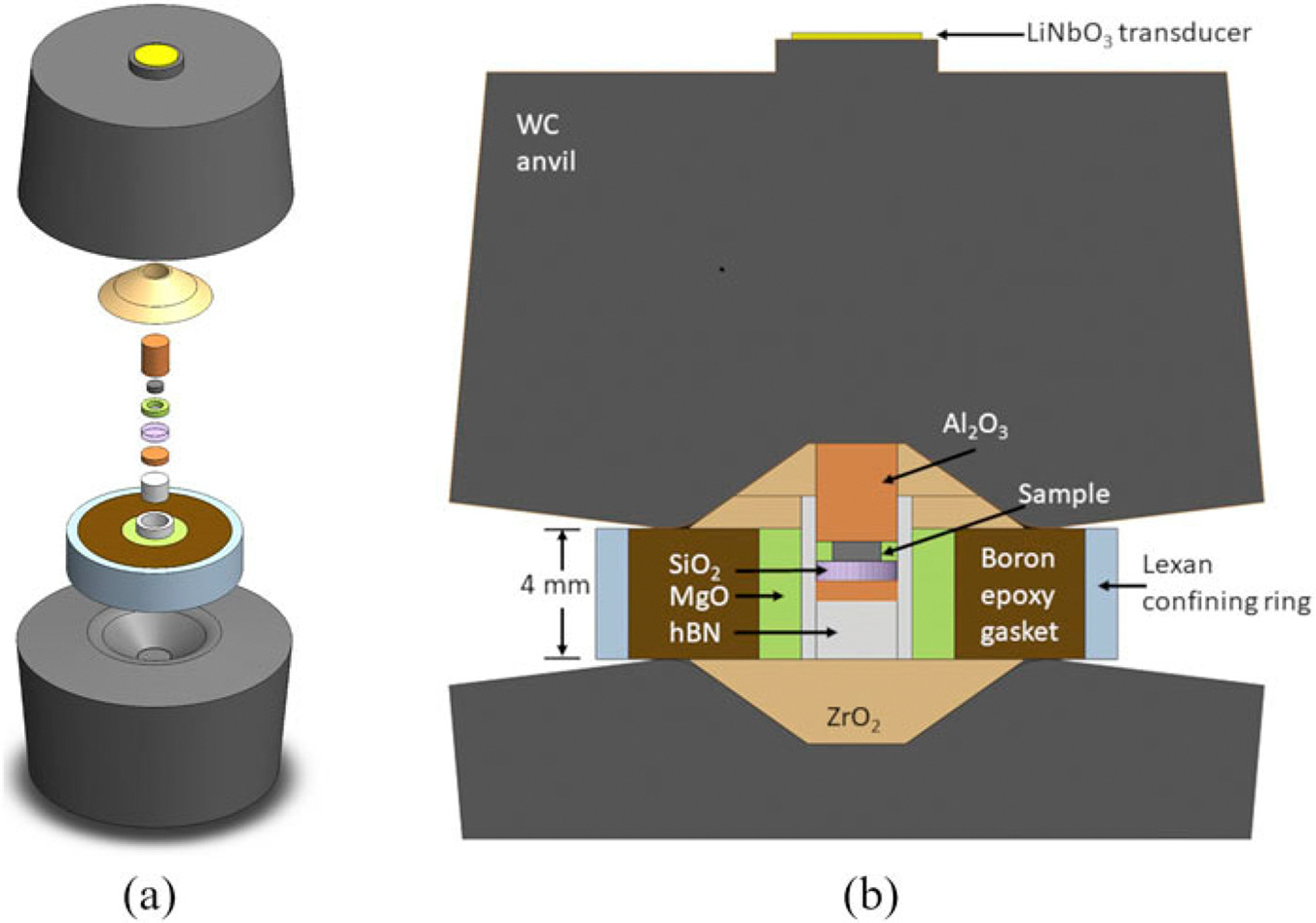
The high pressure inner cell assembly, including sample is shown in (a) the exploding view and (b) in the cross-sectional view.^36^ Reproduced from Husband *et al.*, J. Synchrotron. Radiat. **30** (Pt 4), 671–685 (2023), with permission from AIP Publishing.

The anvil press, also referred to as Paris–Edinburgh press, or LVP, is a method whereby a sample (size ~10 mm^3^) is axially compressed by two anvils, typically of WC. The anvils have a cupped geometry where cups, typically of ZrO_2_, fit to form an inner assembly. Given the LVP geometry, speed of sound measurement is limited to an ultrasonic interferometric technique very similar to the pulse-echo technique described earlier in this review (Sec. II C 1). Ultrasonic sound waves are commonly generated by 10° Y-rotated lithium niobate (LiNbO_3_) single crystal, typically about 2.54 mm in diameter with fundamental frequencies of 50 MHz longitude wave and 30 MHz sheer wave. The ultrasonic wave travels through the WC anvil to an Al_2_O_3_ buffer rod where it then reaches the sample and is reflected back from a backing plate (Cu and more recently SiO_2_ or Al_2_O_3_) to the transducer. A 2.5 *μ*m thick gold foil is commonly positioned on either end of the Al_2_O_3_ to maximize ultrasonic energy coupling. There is a lack of discussion or recognition in the literature if there are any adverse effects of this interface layer with respect to sample purity. Concentric rings of various materials surround the sample to support the sample shape while also providing a quasi-hydrostatic sample environment (hexagonal boron nitride (h-BN)), or act as a pressure marker (NaCl, Pt, Au, and more recently MgO). A boron epoxy gasket and Lexan confining ring are used to enable XRD analysis. XRD can be used to obtain lattice parameters of pressure marker materials used in the LVP assembly. Temperature is usually generated by cylindrical graphite heaters that surround the sample and measured by a thermocouple (type K: NiCr-NiAl, or type C: WRe5%–WRe26%). Historically LVP was limited to solid phase samples as the thickness between the two anvils could not be measured with reasonable accuracy. The limitation of solid phase samples has been overcome by using x-ray radiography measurements to define sample length (Fig. 12).

In 2003, Gauthier *et al.* of the Universite Pierre et Marie Curie presented a LVP instrument that could simultaneously measure sound speed and XRD of a solid phase material, validating the technique at ambient temperature up to 6 GPa with Ge followed by Fe_64_Ni_36_.^111,112^ Measuring the sound speed of solid and liquid phase materials was reported in 2011 by Song *et al.* of the Chinese Academy of Sciences via a heated LVP measuring Na up to 493 K and 2 GPa, followed in 2014 by Sn up to 800 K and 5 GPa.^113,114^ The instrument by Song *et al.* was able to measure liquid samples via a cylindrical WC anvil with a groove where the length dimension change was determined by the WC anvil stress-strain relationship in its elastic behavior. In 2012, Kono *et al.* of the U.S. Department of Energy High Pressure Collaborative Access Team (HP-CAT) built on the reports of both, Gauthier *et al.* and Song *et al.*, to present the first instrument to measure sound speed of SiO_2_ in the solid and liquid state and simultaneously its structure with XRD.^37^ The HP-CAT group has used this set up to study Ge sound speed at 4.5 GPa at temperatures of 973 and 1053 K^115^ and recently materials like multicomponent alloys and silicate melts.^116–118^ In a 2020 collaboration with researchers at Los Alamos National Laboratory, the HP-CAT set up was used to show how implementing a broadband Gaussian wavelet approach led to data acquisition of 1–2 orders of magnitude faster than the traditional single frequency tone burst stepping method.^36^ By leveraging the capabilities of wavelet transforms that provide a localized representation of the signal in both time and frequency domains based on Gaussian functions, researchers could analyze signals obtained from various measurements such as pressure, temperature, or spectroscopic data.

In addition to the advancement of simultaneously measuring speed of sound and crystallinity, the ability to simultaneously measure speed of sound and density in a LVP has been realized. In 2016, Shimoyama *et al.* of Osaka University simultaneously measured the speed of sound and density for solid and liquid Fe-C up to 3.4 GPa and 1850 K.^119^ The LVP design of Shimoyama *et al.* consisted of two sample chambers, a larger bottom sample chamber where speed of sound was measured using the ultrasonic interferometric technique, and a smaller top chamber where a sapphire capsule held sample material for density measurements using the x-ray absorption method. The separation and size difference of the samples within the LVP was necessary as the density measurements were better suited to a smaller diameter sample. Conversely, a larger sample diameter assisted recording speed of sound measurements because it reduced overlapping echoes. In 2019 Terasaki *et al.* of Osaka University expanded the work of Shimoyama *et al.* to include x-ray tomography which enabled three-dimensional information on the sample thickness to provide more precise density measurements of Ni_68_S_32_ to 5.6 GPa and 1045 K.^38^ Simultaneous measurements of density and speed of sound decrease uncertainties and provide datasets with aligned experimental conditions (useful in geophysical applications). Before 2016, simultaneous measurements of density and speed of sound in liquids were only available from shock compression experiments (discussed in Sec. IID 3), and in solid materials via DAC technique.

Anvil presses are commonly limited to 1–25 GPa, but in 2014 Yamazaki *et al.* of Okayama University reported on, in particular, designed sintered diamond anvils that could perform *in situ* x-ray measurements up to a pressure of 109 GPa.^120^ Other continued work on increasing the pressure in an anvil press above the typical upper limit of 25 GPa led to a 2017 report by Ishii *et al.* of University of Bayreuth using WC anvils able to reach 65 GPa.^121^ To our knowledge, these advanced LVP geometries have not been used to measure sound speed of materials. Therefore, further discussion is beyond the scope of this review, but for further information the reader is directed to a 2019 review by Ishii *et al.*^122^

##### b. Diamond anvil cell (DAC).

DAC is a technique that enables measuring the speed of sound for solid and liquid materials at more extreme conditions (300 GPa and 6000 K) than available by an LVP. Here we only briefly discuss DAC design. There is much more literature on DAC, including a 2020 review by Anzellini and Boccato that the reader is referred to for details on DAC that are not covered by this review.^110^ A DAC consists of two gem-quality, single crystal, diamonds that act as anvils. Nano-polycrystalline diamond (NPD) have also been recently used in DAC, but for the XAS technique, not speed of sound.^123^ The diamond anvils have cutlets of varying size, usually 100–600 *μ*m in diameter and about 40 *μ*m in thickness. The anvils are placed above and below a metallic gasket (gasket material: Re, W, Stainless Steel, or Be when lateral optical access is required) and the space between the gasket and cutlets form the pressure chamber. In the DAC’s pressure chamber, the sample (sample volume is typically ~1 × 10^−4^ mm^3^) is loaded with the common addition of a pressure transmitting medium (PTM) and pressure transducer. The PTM facilitates the desired quasi-hydrostatic environment for the sample. There are many considerations for a PTM that will not be detailed here, but one should be aware of the sample contamination that can take place through diffusion or chemical reaction. The pressure gauge included in the pressure chamber is usually a ruby (Cr:Al_2_O_3_) or a Sm-doped Y_3_Al_5_O_12_ sphere. During an experiment, visible lasers measure the pressure-induced shifts in the florescence signal of the ruby sphere acting as a pressure gauge. Pressure is applied to the sample by compressing the diamond anvils via screws or inflating a metallic membrane.

In the past 20 years, advancements for speed of sound measurements in a DAC are similar to those in a LVP, and have included increasing the (a) pressure, (b) sample size, (c) temperature, and (d) simultaneous *in situ* measurements. A higher-pressure range is achieved via advances in DAC geometry, which commonly leads to decreasing sample volume. An example of new geometry for DAC that has fostered growth in the field is the “BX90”, first published in 2012, which reimagined the way pressure screws were located and included four u-shaped cuts in the cylinder part of the cell to allow access for multiple experiment techniques.^124^ Increasing the sample size in a DAC was achieved in 2013 by Boehler *et al.* of the Carnegie Institution of Washington with 1 mm cutlet anvils for 100 times greater sample size (~2 × 10^−2^ mm^3^) up to 90 GPa. Realizing pressures far above 100 GPa with a large sample size remains challenging.^125^ To overcome the challenge of a 100 GPa upper-pressure limit with a large sample size Kono *et al.* of the Carnegie Institution of Washington combined a Paris–Edinburgh (PE) press and a DAC to reach 131 GPa and study the structural evolution of SiO_2_.^126,127^ A detailed study in 2018 by O’Bannon *et al.* of Lawrence Livermore National Laboratory showed that generating static pressures over ~300 GPa in a DAC would require fundamentally different geometries than the three (bevel, double bevel, cutlet) currently in use.^128^ An example for a fundamentally different geometry is presented in the 2018 paper by Dewaele *et al.*^129^ The CEA’s military applications division in France used new toroidal DAC geometry that extended the range of DAC to ~600 GPa to study gold, aluminum, potassium chloride, and argon samples. The work by Dewaele *et al.* outlines five experimental runs with 5 *μ*m sized samples at ambient temperature conditions and pressures ranging from 317 to 617 GPa.^129^ Research to achieve even higher pressures with double-stage DACs is reported in articles by Dubrovinskaia.^130–132^

It should be noted that the pressure measurement in a DAC remains the largest contribution to DAC speed of sound measurement uncertainty. For experiments at P < 100 GPa the internal pressure gauge of ruby (Cr:Al_2_O_3_) or Sm-doped Y_3_Al_5_O_12_ spheres with an uncertainty of ±0.1 GPa – 0.2 GPa (0.2%–10%) is commonly used, but care must be taken not to use them outside their calibrated region due to propagation of even larger uncertainties.^72,133^ For experiments where P> 100 GPa, the ruby signal is wider and weaker and so a Raman signal obtained from the center of the diamond cutlet is preferable. Akahama and Kawamura of the University of Hyogo completed extensive research on calibrating the Raman signal for a DAC from 2002 to 2010 with the highest pressure calibrated at 408 GPa leading to an uncertainty at 1–140 GPa of ±0.66 GPa, 100–310 GPa of ±8 GPa, and at 265–408 GPa of ±6 GPa – 9 GPa.^134–136^ In practice, it was found most reports simply state a 3% uncertainty for pressure measurement via Raman signal without explanation as to how that conclusion was drawn. In parallel, efforts were made to use *in situ* XRD data of the PTM to empirically relate the evolution of the lattice parameters to the equation of state (EoS) of the material.^137^ XRD and EoS are used together to define sample pressure when the most extreme temperature conditions are realized in a DAC (enabled by laser heating), which limits spectroscopic techniques only executable before and after heating. The EoS creation for pressure sensing materials was commonly completed using speed of sound data measured using ultrasonic interferometric and shock wave techniques.^138^

Advancement in DAC sound speed measurements is experiments being performed at elevated temperatures by either external resistive heating, internal resistive heating, or laser heating. External resistive heating in DAC requires the entire DAC assembly to be uniformly heated by Joule effect, but at the cost of large thermal dispersion and mechanical integrity leading to a lower pressure operating range.^139,140^ Temperature in an external resistive heated DAC is usually measured by a thermocouple placed at the gasket-sample interface near the center of the high-pressure cell. The type of thermocouple is commonly not disclosed, but when specified they are typically type K: NiCr-NiAl, type S: PtRh10%-Pt, or type R: PtRh13%-Pt with uncertainties of ±1.5–5 K.^141^ The use of internal resistive heating in a DAC confines the increase in temperature within the pressure chamber, allowing for higher temperatures to be achieved. This localized thermal load primarily affects the sample, thereby mitigating potential adverse effects on the overall integrity of the assembly. Temperature in an internal resistive heated DAC can be measured by thermocouple, as described for external heated DACs, or with a spectroradiometric method with an uncertainty of ±100–900 K (±10%–15%).^142^ By using a high-power laser (near-IR: Nd:YAG, Nd:YLF, orthovanadate crystals, or CO_2_) the most extreme experimental temperatures can be reached in DAC.^143,144^ Measuring temperature in laser heated DAC is complex and always needs to be critically evaluated. The reader should refer to the 2020 review by Anzellini and Boccato for more specific details.^110^ Simply put, temperature in a laser heated DAC is usually measured using a spectroradiometric method leading to an overall combined sample temperature uncertainty of ±100–900 K (±10%–15%). In the case of any spectroradiometric method, routine calibration is performed to a known standard, like those available from the U.S. National Institute of Standards and Technology (NIST). To be clear, none of these elevated temperature measurements are new developments from the past twenty years; internal heating was first disclosed in a DAC by Lui and Bassett in 1975 and laser heating was first completed by Ming and Bassett in 1974.^145^ Nevertheless, more experiments are being conducted at elevated temperatures to measure the sound speed of condensed matter.^146,147^ In our review of the recent literature, few studies focused on investigating or proposing a solution to large temperature measurement uncertainties within DAC. One exception is a 2019 report by Sinmyo Hirose and Ohishi with their study of iron’s melting curve. Their work included XRD and no speed of sound measurements so we still find the pursuit of lower uncertainties and better metrology in DAC an underexplored area with great opportunity for growth.^148^

Another main advancement has been the use of advanced beamlines resulting in decreased timescales for acoustic measurements. Within a DAC sound speed has been measured using (1) ultrasonic interferometric methods, (2) Brillouin scattering, and most recently (3) IXS and (4) picosecond acoustics techniques. Li and Liebermann reviewed ultrasonic interferometric DAC methods in 2014.^25^

**FIG. 13.**
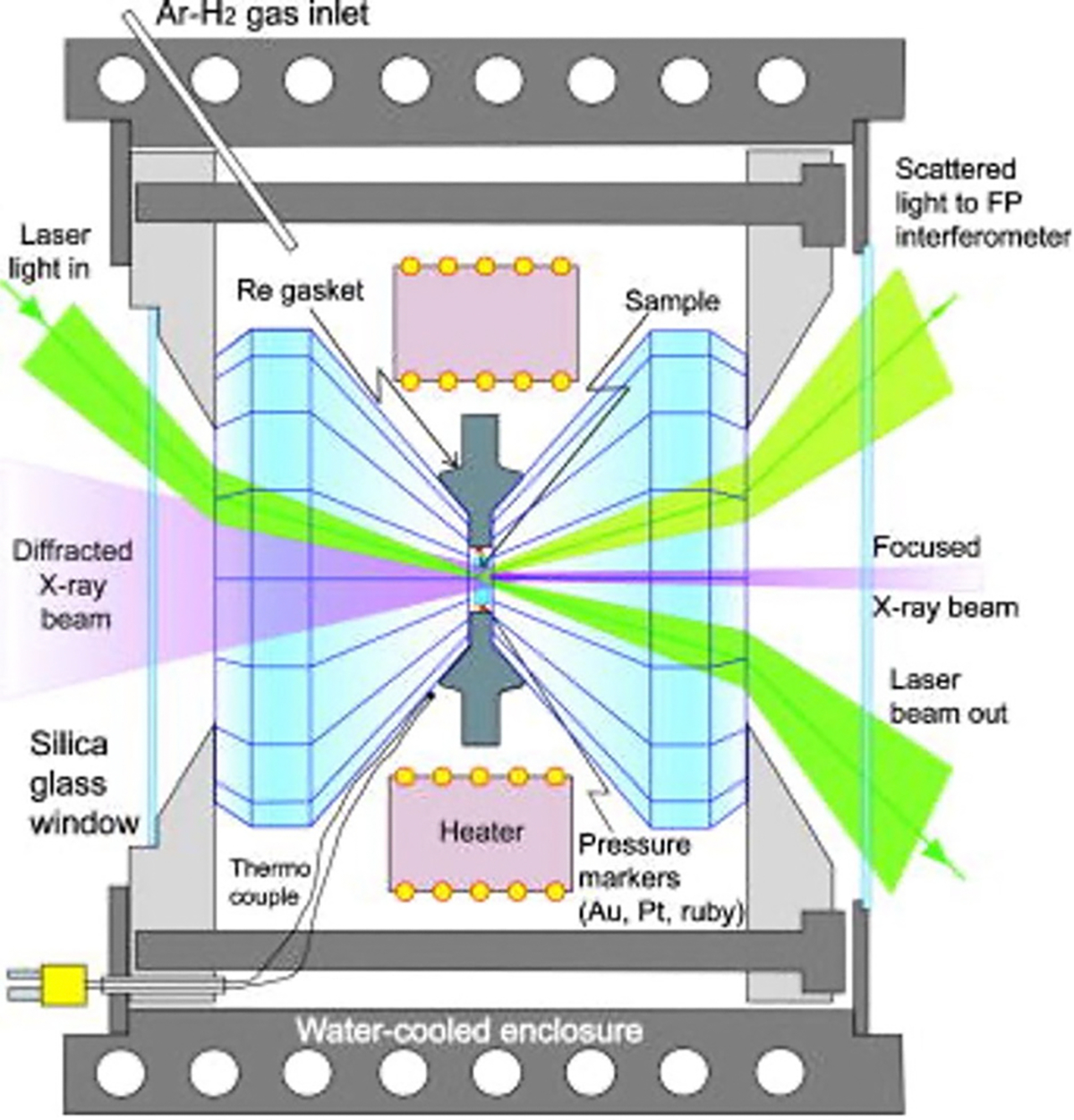
Schematic of the heated diamond anvil cell apparatus of Sinogeikin *et al*. at Argonne National Laboratory user facility to measure the speed of sound using Brillouin scattering and density by x-ray diffraction.^150^ Reproduced from Sinogeikin *et al*., Rev. Scient. Instrum. **77**(10), 103905 (2006), with permission from AIP Publishing.

For details on Brillouin scattering in diamond anvil cells prior to 2003 the reader is directed to a review by Polian.^149^ In 2006 Sinogeikin *et al.* at Argonne National Laboratory user facility reported on a resistively heated DAC developed to study sound speed using Brillouin scatting.^150^ In 2009, a group of researchers from Japan, Murakami *et al.*, reported a new IR laser heated DAC to study perovskite materials via Brillouin scattering.^142^ Members of the Murakami *et al.* paper had collaborated in 2007 with researchers from University of Illinois on sound velocity of perovskite^151^ using a method first reported in 1998^152^ from University of Illinois and ETH Zürich with Brillouin spectroscopy. Since the initial presentation in 2009 Murakami *et al.* have published on further work on perovskite materials at temperatures up to 2700 K and pressures up to 124 GPa (Fig. 13).^153^

Inelastic x-ray scattering spectroscopy measures the dynamic structure factor, *S*(*E*,*Q*), that contains information on all electronic excitations and can be used to measure the speed of sound in a solid or liquid material. A 2016 review by Shen and Mao lays out specific methods and advances in x-ray analysis of materials in DAC.^154^ The main drawback of IXS experiments is the dependency on a synchrotron facility which makes it less accessible compared to laboratory-scale techniques.^155,156^ Overall IXS as a method to measure speed of sound for materials is actively pursued to study condensed phase materials like Fe, Ni, and silicates, mostly of interest in geological sciences. For example, in 2017 using the beamline BL35XU, SPring-8 (first operation in 2000^157^) Kawaguchi *et al.* employed high-resolution IXS to measure the speed of sound for liquid Fe-Ni-S in both, resistive, and laser-heated DAC.^158^ In 2020, Kuwayama *et al.* used a LH DAC IXS spectroscopy on a beamline at the same facilities and reported three sound speed measurements of liquid phase Fe at 2200 K/16 GPa, 2200 K/32.7 GPa, and 2700 K/44.9 GPa.^159^ Kuwayama *et al.* suggested an updated EoS for Fe based on the three sound speed measurements, along with 11 density measurements. Also in 2020, Nakajima *et al.* performed IXS experiments on the same laser heated DAC used by Kuwayama *et al.* to report six liquid-phase sound speed measurements on Fe–Si alloys from 2760 to 3270 K and 27 to 56 GPa.^160^

**FIG. 14.**
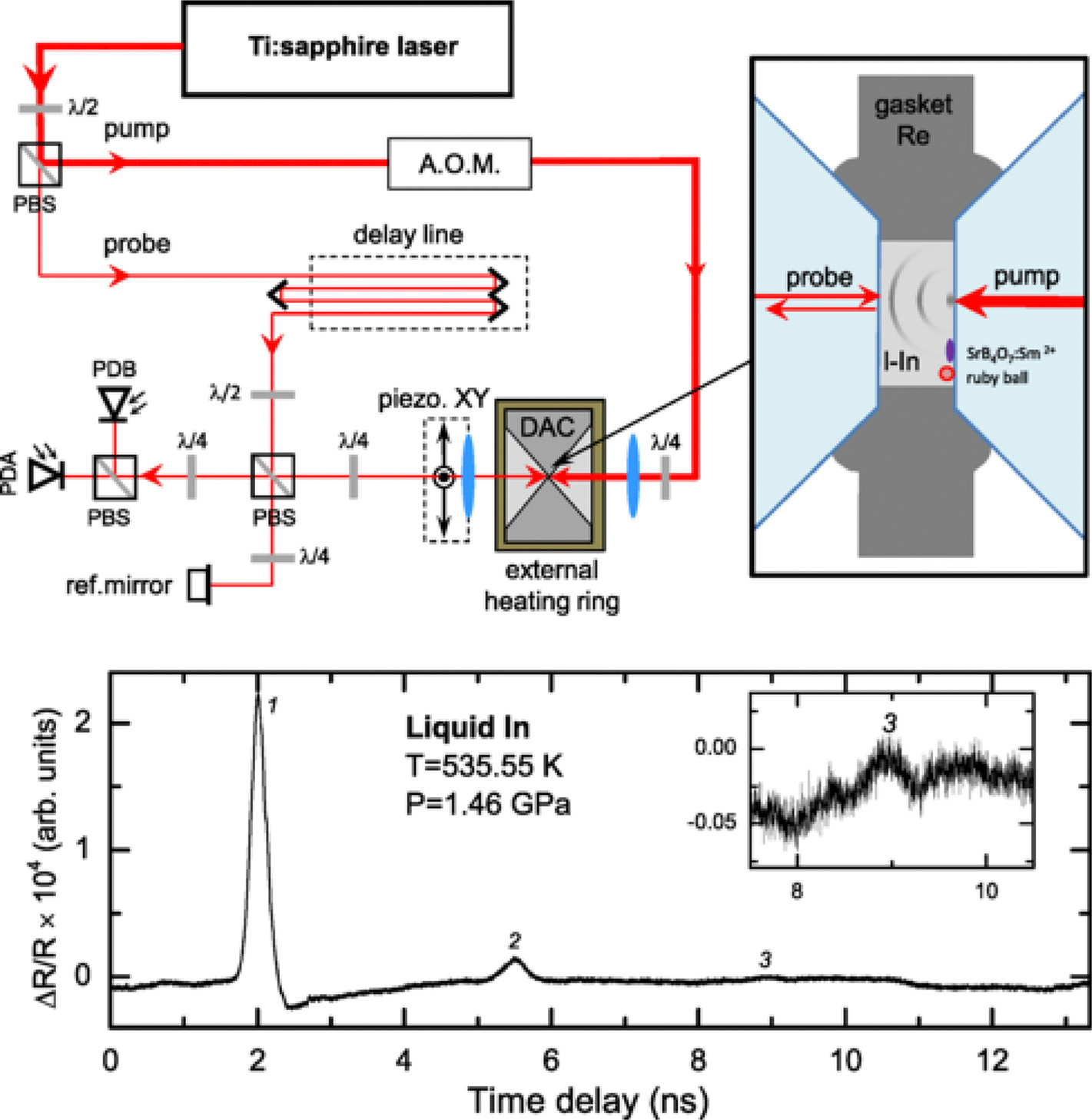
Schematic of the heated diamond anvil cell apparatus of Ayrinhac *et al.* to measure the indium melting curve by speed of sound measurements using the picosecond acoustics method.^164^ Reproduced from Ayrinhac *et al.*, Phys. Rev. Mater. **6**(6), 063403 (2022), with permission from AIP Publishing.

Picosecond acoustics (PA) is a time-resolved optical method that uses a pump-probe process to generate and detect propagating strain waves in condensed matter materials. A 2022 review by Boccato *et al.* goes into detail about the picosecond acoustic method to measure speed of sound at extreme conditions.^161^ Some key publications include the first dissemination of the method for condensed phase materials in a DAC by Decremps *et al.* of the Universite Pierre et Marie Curie in 2015, then the later use of PA in a resistively heated DAC to study cesium along a 500 K isotherm from 1 to 4.5 GPa.^162,163^ The same group has continued using the apparatus to measure an array of materials including the indium melting curve from 420 to 680 K from ambient pressure to 6 GPa, stating that the technique outperformed other methods and could be used to enable more precise EoSs (Fig. 14).^164^

Beginning in 2021 developments to combine a DAC and shock compression to study the solid–liquid phase boundary of materials at extreme conditions started, but the setup at this time used XRD to probe the atomic and molecular structure of materials, not SoS, thus it is not detailed in this review.^36,165^

#### 3. Shock compression: 5–600+ GPa

Shock compression is a dynamic technique to measure a material’s sound speed at higher pressures than available in the static techniques with a pressure vessel, LVP, or DAC. Compared to DAC, shock compression allows for (1) higher pressures, (2) lower background signal, and (3) solid and most recently liquid phase material investigation.^166^ This experimental approach provides valuable insights into the dynamic behavior of materials, allowing for a better understanding of their elastic properties and response to high-pressure conditions. In shock compression experiments, the speed of sound is measured using various techniques that take advantage of the rapid compression and subsequent release of materials under high-pressure shock waves. Briefly, shock compression involves high velocity mechanical impact, explosives, or high-power laser arrangements to ultimately generate a shock wave that propagates through the material of interest.

**FIG. 15.**
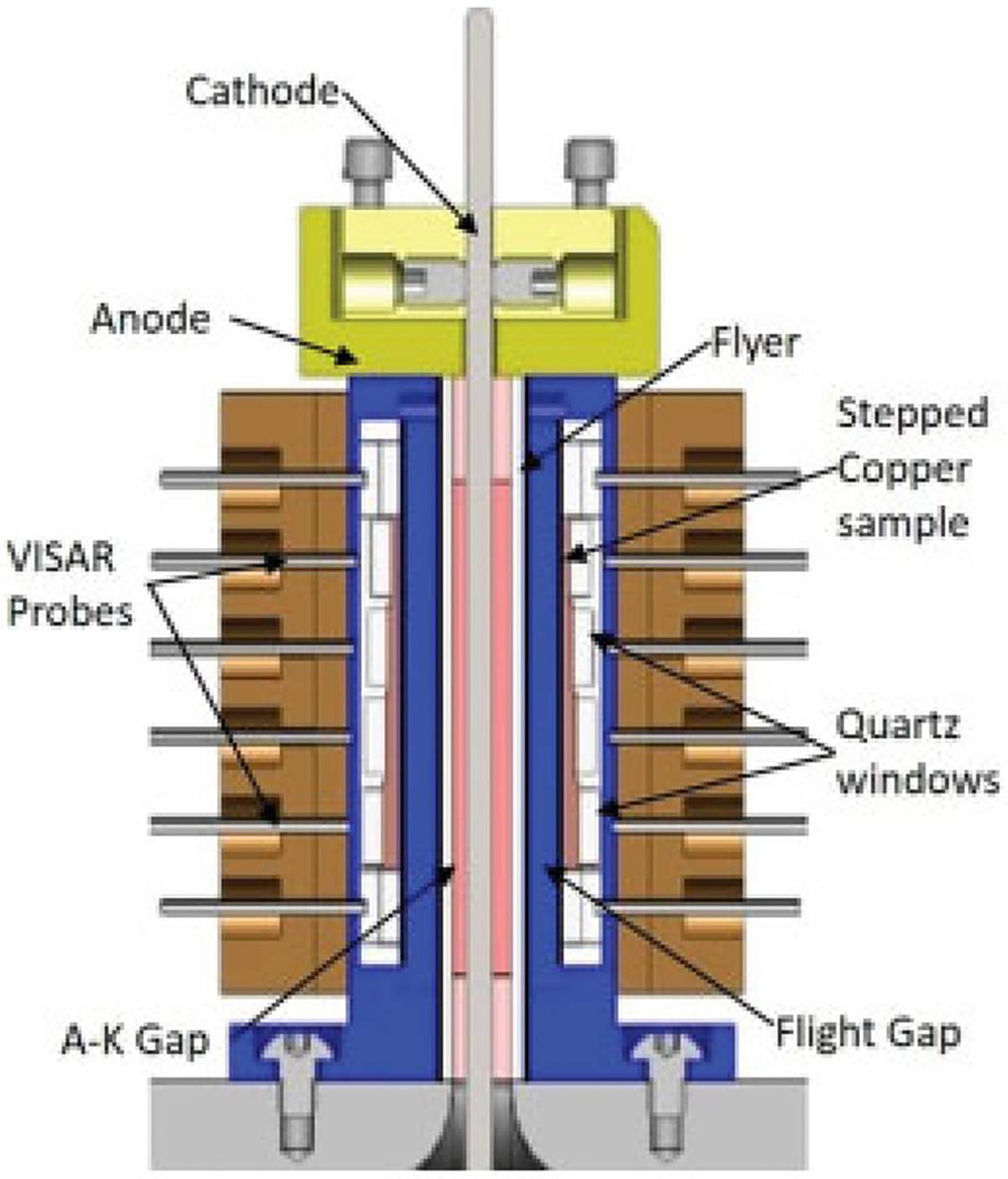
Schematic of shock wave apparatus used by McCoy *et al*. to measure liquid copper speed of sound up to 400 GPa.^220^ Reproduced from McCoy *et al*., Phys. Rev. B **96**(17), 174109 (2017), with permission from AIP Publishing.

Two common methods to measure SoS via shock compression are time-of-flight and velocity interferometry. In the time-of-flight technique a sensor, such as a piezoelectric transducer, is placed at a known distance from the material being studied. When a shock wave passes through the material, it compresses and generates a high-pressure region. As the shock wave propagates, it also creates a compressional wave, or a sound wave, within the material. The transducer detects the arrival of this sound wave, and by measuring the time it takes for the wave to travel the known distance, the speed of sound can be calculated. The velocity interferometry method utilizes laser interferometry to measure the velocity of material particles as they undergo shock compression, Fig. 15. By analyzing the Doppler shift in the frequency of laser light reflected from the material, the particle velocity can be determined. The SoS is then calculated based on the ratio of the particle velocity to the density of the material. An important descriptor in this field is the Hugoniot curve. The Hugoniot curve represents the relationship between the material’s shock velocity and its particle velocity. It is derived from shock compression experiments and provides valuable information about a material’s SoS under extreme conditions. At the initial stages of shock compression, the shock wave rapidly compresses the material, leading to an increase in its density and pressure. The Hugoniot curve can reveal important information about phase transitions, structural changes, and other dynamic properties of the material under extreme conditions. A more in-depth review on the theory and the field of shock compression experiments can be found in a publication by Davidson and Graham^167^ and a book from RA Graham.^168^

Important milestones in shock compression experiments over the past 20 years include the development of advanced diagnostic techniques, such as ultrafast imaging and spectroscopy and enabling real-time visualization and characterization of shock-induced processes. Additionally, the advent of laser-driven shock wave generators has expanded the range of materials and pressures that can be studied. Single, dual, and multi-phase techniques have been reported in the literature with the number of phases in the apparatus being dependent on the target pressure range. An example is the work by Smith *et al.* at the National Ignition Facility where a laser driven pressure wave was used to reach pressures up to 1.4 TPa to study pure iron.^169^ Until recently, shock compression only measured solid-phase sound speed. The limitation to solid phase was because XRD could not obtain quantifiable high quality information of liquid phase sample due to limited photon flux in x-ray sources. Shock compression of liquids has been studied for noble gases and organic compounds, but those compounds are outside of scope for this review.^170^ Reports starting in 2015 by Briggs *et al.* demonstrated the ability to study differences between solid and liquid phase materials with shock compression due to x-ray free electron lasers (XFEL) for Bi, followed by Sc and Sn.^171–173^ The reported uncertainty for the Sc study by Briggs *et al.* in shock speed was estimated at ~4% and pressure ~7% by propagating through the Rankine–Hugoniot equations. See a 2016 review by Bostedt *et al.* for more details on LINAC coherent light sources.^174^ In experiments by Briggs *et al.* velocimetry measurements were collected at the Sn/LiF interface using a 1D VISAR system first reported in 2004.^175^ Experimental pressures were determined using impedance matching between the Sn^2–4^ and LiF EoS with refractive index correction.^176,177^ Additional research to understand shock melting in pure elements includes work by Jensen *et al.* and Hixson *et al.* for cerium.^178,179^ Work on other pure elements has also expanded and is outlined in Table II. In addition to pure materials being studied by shock compression, multicomponent materials have had increased investigation like in the case for SoS of lithium fluoride, LiF, due to its popularity as an optical window in dynamic compression experiments. Wallace *et al.* applied the experimental configuration by Liu *et al.*^180^ to study single crystal LiF, and their results disagreed with Liu *et al.* that melting occurred between 134 and 152 GPa.^181^ The findings motivated the group to build upon their initial report with Hawreliak *et al.* measuring LiF up to 230 GPa which has provided more measurement data, emphasizing the need for a multiphase EoS.^73^ Ultimately, through the progression of experimental advancements in shock compression studies, the combination of computational modeling and simulations has led to a more all-encompassing grasp of shock compression phenomena. The detailed examination of simulations related to high-pressure experiments is reserved for Sec. IV.

### E. Respecting a method’s goal, respecting limitations, and defining “good enough”

Measuring the SoS in condensed matter under extreme conditions necessitates a deep understanding and respect for the limitations inherent in various experimental methodologies. Each method has its own unique strengths and weaknesses tailored to specific aspects of such a complex measurement. Some methods might excel in high-temperature scenarios, others in high-pressure situations, while a few might be geared toward specific phases of matter such as solids or liquids. As scientific tools and techniques stand currently, no single experimental method can seamlessly span all these parameters simultaneously with equal precision and accuracy. It is paramount that scientists recognize these limitations, adjusting their experiments accordingly to avoid inaccurate measurements or misleading conclusions. Ultimately, the choice of method should align with the specific goals of the experiment, whether it is to gauge the speed of sound in solids at high temperature, or liquids under extreme pressure, acknowledging that each experiment contributes a piece to the larger puzzle of understanding sound propagation in extreme conditions.

Interestingly, there can be instances of overlap between different techniques, such as in the case of diamond anvil cell and shock compression experiments. Both methods overlap in pressure at around 100 GPa, serving as a potential common ground for cross-verification of results. This overlap can help build a more complete and accurate picture of the properties of matter under these extreme conditions, thereby advancing our understanding in this field. Such a positive outcome can only be achieved if honest discussions of the benefits and limitations of differing techniques are held.

On the note of different measurement techniques, current SoS instruments available to measure condensed matter at extreme high temperatures and pressures are highly specialized and custom-built. As a result, the process of obtaining data are slow and expensive, leading to less data and limiting the rate of scientific progress in this area. Given the current scarcity of commercial SoS instruments for measurements at extreme conditions, it is understandable why a lack of standardized measurement protocols prevails in this field. The ASTM Committee E07 on nondestructive testing and its subcommittee E07.06 has published the standard ASTM E494–20 for measuring ultrasonic velocity of a material by the pulse echo method; but this standard does not include materials at elevated pressure or temperature conditions.^187^ Most research groups studying materials at extreme conditions develop their custom methodologies, with parameters calibrated to their specific requirements. However, as the demand for such measurements increases and the potential for commercialization of these advanced techniques becomes increasingly apparent, the need for uniform measurement standards increases. Standardizing the methodologies not only ensures the accuracy and consistency of data across different setups, but it also aids in data replication and verification, making research more robust and credible. As we anticipate a future where these techniques become commercially available, the scientific community must take proactive steps toward the development of universally accepted measurement standards to advance this field more effectively and uniformly.

The debate on material characterization data accuracy is complex. Funding agencies aim to avoid diminishing returns on investments by reducing data uncertainties, a logical approach to reallocating funds for greater returns once a limit is reached. Industry shares this perspective, and it is essential to decide if the goal is addressing immediate issues or advancing knowledge for its own sake. Scientists also consider the practical application of their work. However, the full extent of data potential remains uncertain. Conversely, a limited comprehension of the significance of prioritizing high-quality measurements can be perilous because (1) the essence of scientific pursuit is to consistently push the boundaries, and (2) high-quality reference measurements can, in fact, reduce the total number of measurements required to accurately characterize materials. For example, pursuing low uncertainties, like SoS to 0.01% uncertainty, may seem excessive if considered by itself. However, low uncertainties in properties like SoS relate to other properties and thus fewer measurements overall are needed to characterize the material.^19^ Advanced thermodynamic modeling using equations of state (EoS) has successfully extended the understanding of material behavior beyond the realm of experimental data by focusing on fewer but more accurate measurements.

In summary, there are many facets to designing a measurement technique for the SoS in extreme environments. Innovation should consider what has previously been done and then take advantage of advances in peripheral components. Furthermore, innovation should seek to reduce uncertainties of measurement in methods from different perspectives that have not been explored. The well-heeded notes by Australia’s chief scientist, Alan Finkel, in 2019, “People respond to incentives. Change will come only when grants and promotions are contingent on best practice.”^188^

## III. DATA AVAILABILITY

Currently, data on liquid metals are sparse, and of the that, notably large uncertainties constrain their applied usefulness.^79^ It should be understood that the gap in accurate data exists for the most commercially used pure metallic elements such as aluminum and nickel, and some alloys including bronze and stainless steel.^189^ This knowledge gap stalls the development of new processing methods like additive manufacturing or laser welding across government, academic, and industrial organizations.

A path to overcome this problem is more measurements but the constraints of extreme environments drastically limit the applicable measurement methods. Most experimental setups are developed, built, and maintained in dedicated research facilities and for many properties commercial instruments are not available or only cover a much narrower, limited parameter space. An overview on commercial instruments is beyond the scope of this paper, nevertheless, the importance and impact of commercial instruments for lowering the barrier of entry to report on material properties must be emphasized. Using ionic liquids as an example, a recent review by Dzida *et al.* identified that over 75% of experimental results for the speed of sound in the literature use one instrument, the DSA 5000 by Anton Parr.^190^ This shows that one path to increasing the amount of data collected for a set of properties/materials is a lower barrier to entry by making measurement instruments commercially available. In any case, enabling the ability to measure data cannot get confused with producing reference quality data. Even at conditions that are not extreme, accurate material property measurements require expertise, traceability through calibration, and a rigorous uncertainty estimate.^191^ A recent IUPAC technical report on good reporting practices for measurements highlights the related problem of reporting those measurement results. Only by following good reporting practices and including all metadata, uncertainty estimates, and other relevant information to fully describe the dataset, as exemplified in that report, these valuable data have the appropriate quality to be included in databases for wide user access.^12^

### A. Types of data and databases

Once material properties are measured the data must be made available and to this date the most common path is to publish it in a journal article. More and more initiatives focus on data management plans and encourage other paths to enable user access to original data. Established options include uploading the published data directly into various repositories with persistent links or to submit structured data files as supplementary material with the published articles. The same processes are established for data from *ab initio* simulations, independent of properties.

The data themselves, in the case of this review, Speed of sound data, can be grouped into three categories: (1) measured, (2) derived, and (3) extrapolated. Measured data are obtained by experiments using methods such as those described in Sec. II. Extrapolated data are obtained from theoretical modeling or simulations like an equation of state, CALPHAD, or artificial intelligence (AI) and machine learning (ML) methods. In addition to the best estimate of a value it is also important that it is accompanied by a measure of quality, often expressed as measurement uncertainty. While the methodology to do that is widely established for data from category one,^46^ it is still an active field of research for values from categories two and three. Recognizing the category data are in before using it for simulations that guide design decisions is therefore crucial to avoid potentially catastrophic failure.

Like the data themselves, databases too can be categorized to aid in understanding the type of data being offered and inform the user of the database as to the limits and precautions to take when using data organized in the collection. Three categories of databases are: (1) collected, (2) calculated, and (3) curated. A not-exhaustive list with examples for databases covering multiple fields and purpose is given in Table III. When input and user contributions are collected and organized into a database without further action that verifies quality then the label (1) collected is most appropriate. This also applies if the information is otherwise crowd sourced or accumulated via a web crawler in an automated way. When the stored property values are the result of some sort of calculation then (2) calculated is used as a descriptor. Schemas used in those cases often also accommodate model parameters and not only the results of the calculations. To be listed in the third group, (3) curated, much more effort must be put into the curation of the data collection. Not all repositories are created equal in this space either but they often include multiple steps and additional information that are intended to improve the data quality, such as the provenance of the data the complete bibliographic information from where the data were captured; a quality check if the capture was correct and if the data conform to the expected data group; additional metadata that are needed to interpret the data values correctly, e.g., a reference temperature or pressure for a property like relative linear expansion; a quick check if the reported data are reasonable in the context of what it is reported for – an order of magnitude check; an uncertainty statement from the original author or an estimate from a subject matter expert. There can be many other steps but all of them have in common that they are typically very time intensive, require attention of a professional with appropriate scientific expertise in the focus area of the database, and cannot yet be performed in a similar manner by an automated system.

We leave it to the interested reader to explore the enormous breadth of information reported in the listed repositories. For this review we limit the scope to one example, a resource that is curated inhouse, the NIST Alloy Database.^192^

### B. NIST alloy database

The NIST Alloy Database is a repository of published experimental data with a focus on metallic elements and their binary and ternary alloys.^10,192^ This effort is based in the Thermodynamic Research Center (TRC) at the United States Department of Commerce National Institute of Standards and Technology (NIST). All data are curated by a subject matter expert and are presented as they were reported by the author in the original publication. Thermophysical properties in scope of this repository include speed of sound, density, specific heat, viscosity, enthalpy, phase transition temperatures and many more. Independent of how the data are reported, whether in a tabular format, as equation or in a graphical format, they are captured with provenance, relevant metadata, and a clear statement regarding their quality, quantified in statements of uncertainty.

Easy free access is available through a web application (https://trc.nist.gov/metals_data) that supports a simple search on a graphical interface outlined as periodic table, or an advanced option that allows more complicated queries and offers additional data reduction parameters. Programmatic access is supported through an API, which unlocks the full potential of using electronically available property data in a well-structured data format. Queries in the NIST Alloy Database can not only deliver data sets for properties of a particular element or system of compounds but can also be used to recognize data gaps, and aid new material development.^193^ Using this tool, it was found that only eight captured references report the SoS of liquid zinc, the most recent reported in 1993 by Tsuchiya *et al.* and only six data sets have been recorded for SoS measurements for liquid aluminum, the most recent one published in 1982 by Tsu *et al.*^85,194^ The only two articles including a single datapoint for SoS of liquid titanium each were published in 1984 by Casas *et al.* and Said *et al.* in 2006. In both cases the uncertainties for the methods used were reported with ±10%, thus offering little actionable insight.^195^

**FIG. 16.**
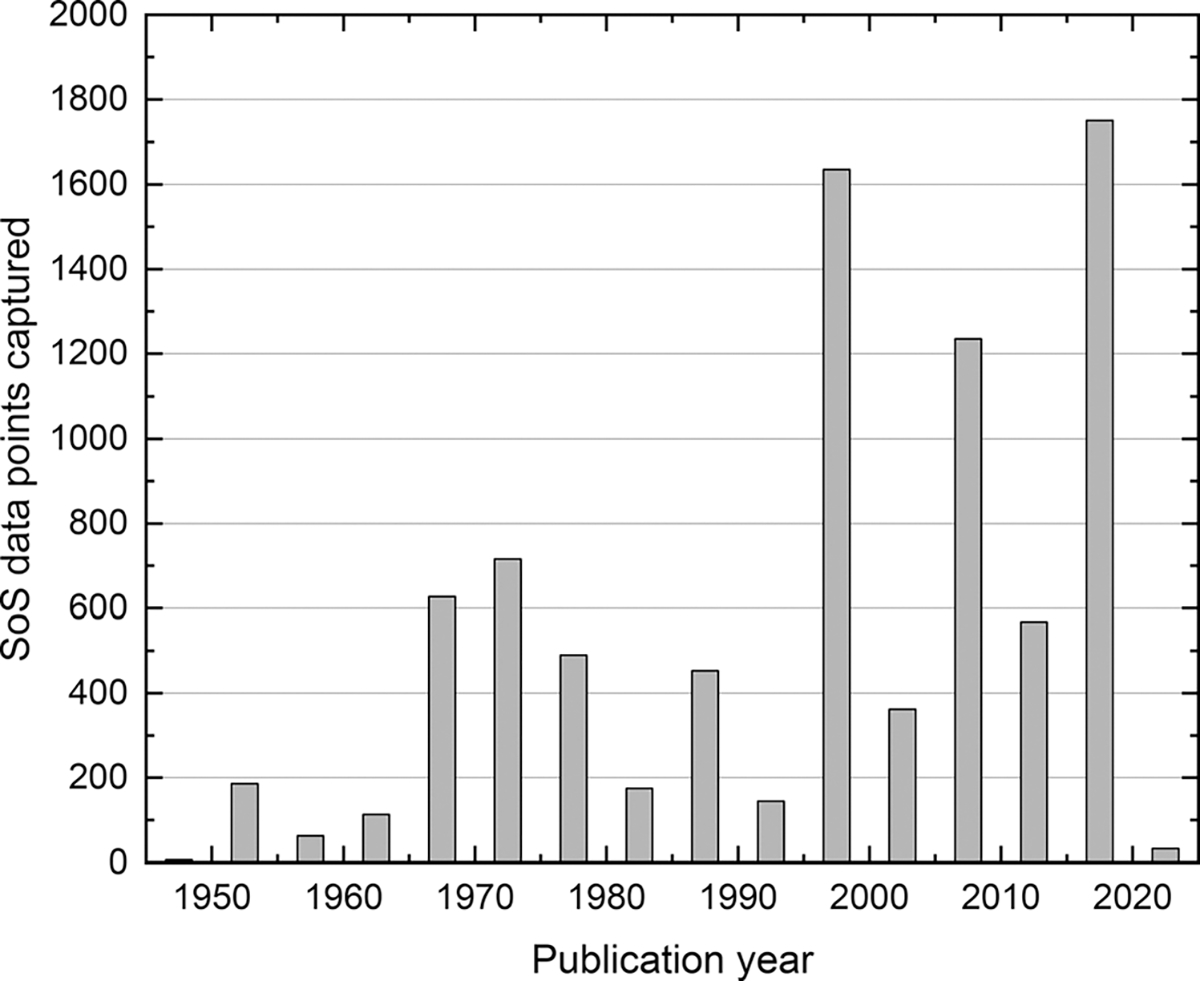
Histogram for speed of sound data points from articles captured in the NIST alloy data repository for elements in scope vs the publication year. Each bar represents a time span of five years (e.g.,: from 1995 to 1999 a total of 1635 SoS data points were captured).

A review by Blairs^23^ published in 2007 presented a great overview for SoS data in pure liquid metals and metalloids. It exemplifies the very limited data coverage for the liquid phase and even more for elevated pressures. The clear advantage of a continuously updated repository is highlighted by the histogram in Fig. 16, which shows how many additional data points were added in the years after Blairs’ review paper. Those extra data points are now also available on the same platform and can be considered when revisiting the correlations for the datasets.

The database contains approximately 25,800 data points for SoS vs pressure and temperature at the time of publication. The values for speed of sound range from some hundred up to about 6000 m/s with only very few data sets above that value.

**FIG. 17.**
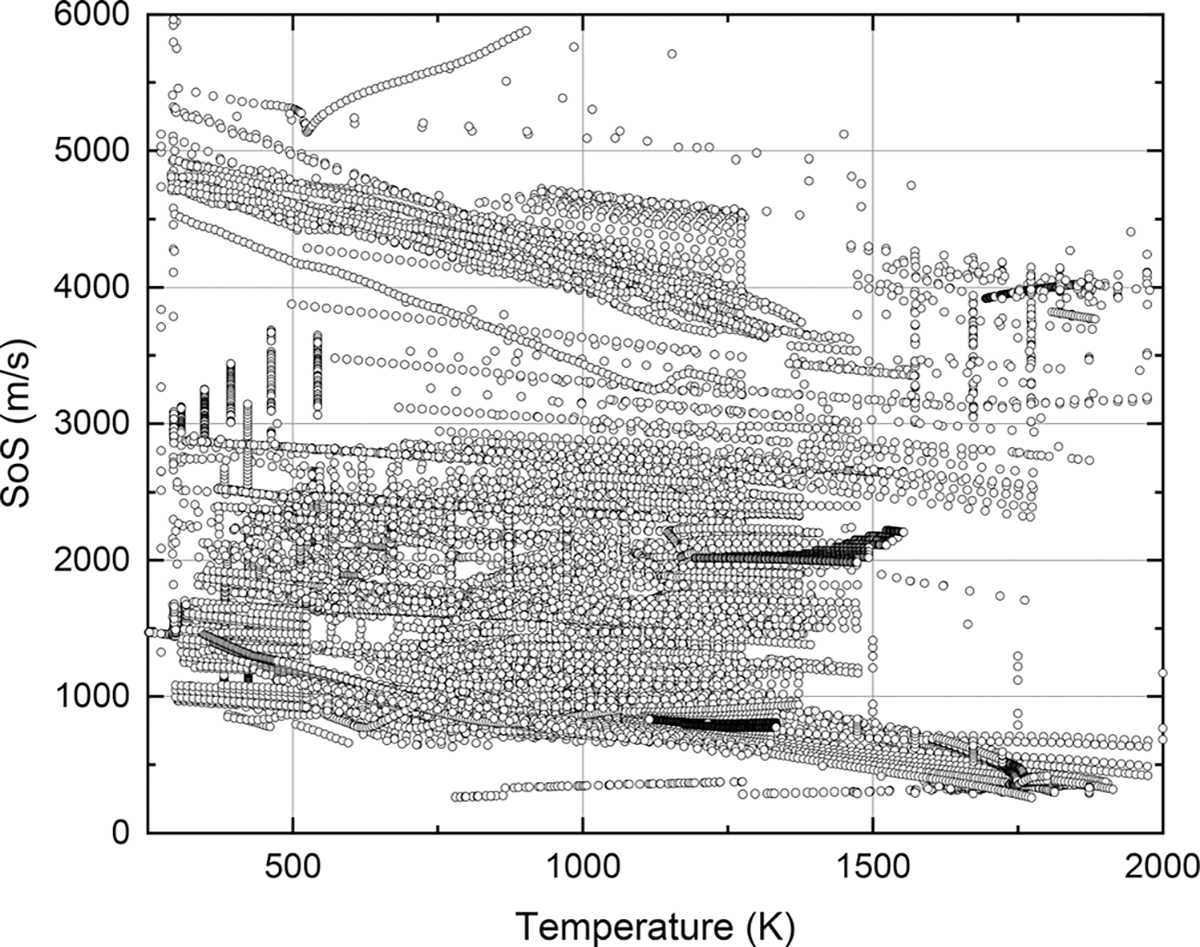
Plot of speed of sound values vs temperature for pure elements and alloys.

**FIG. 18.**
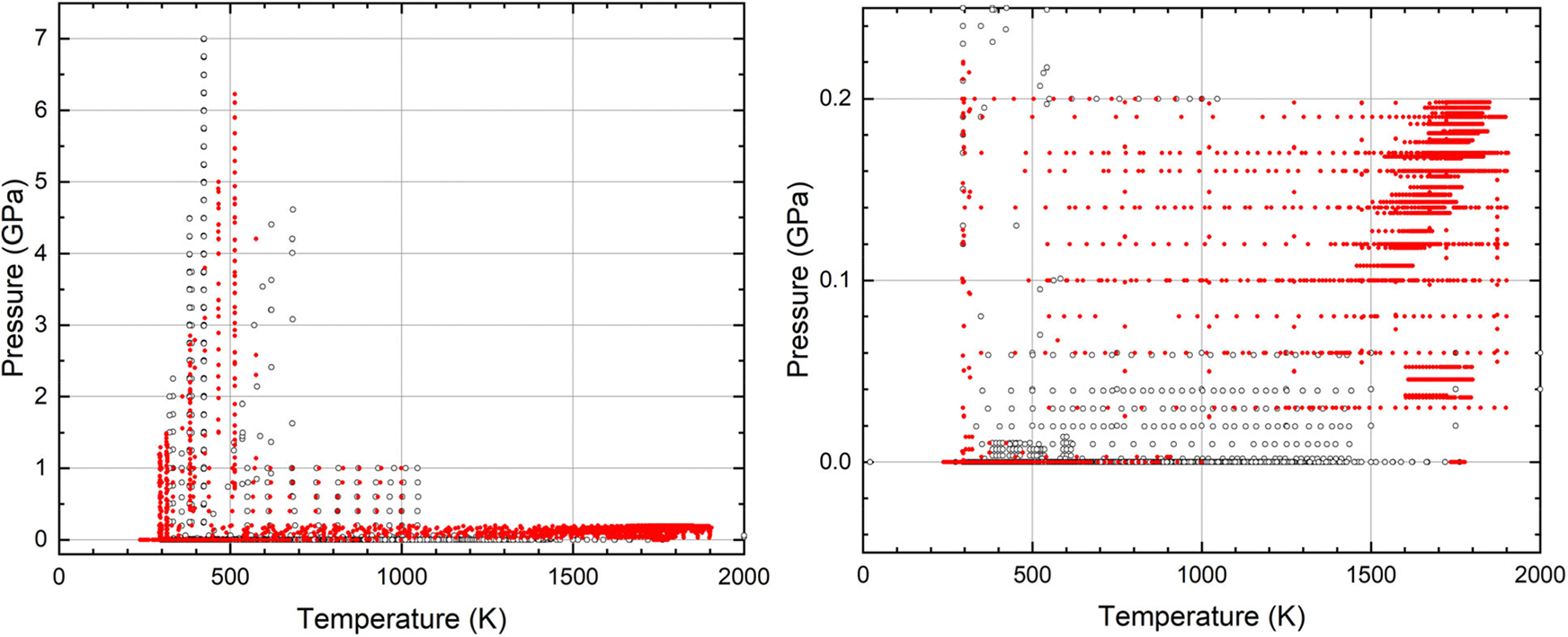
Plot of Speed of Sound data for elements with a melting point below 1000 K as captured in the NIST Alloy Data database. Data points in red are from measurements on Hg alone.

Figure 17 shows all those points in the solid and liquid phase and how fast data availability drops once the temperature exceeds 1500 K. In that whole point cloud, only 3800 reported data points are measured above atmospheric pressure (14.7%) and a total of 153 data points are above a pressure of 7 GPa. Only about 15% of the available data are for pressures above 7 GPa or temperatures above 1000 K. The pure elements with a melting point below 1000 K (elements in scope) represent 7465 of the points and of those 2775 values (37%) are from Hg alone. Of those, excluding Hg, only 1300 (28%) are measured at pressures above atmospheric pressure. These data show that more high-pressure measurements are reported for elements with a lower melting point than with a melting point above 1000 K, which points to the experimental difficulties realizing high pressures simultaneously to maintaining the metals at high temperatures. Figure 18 gives a graphical summary of the data point distribution for pure elements with *T*_m_ ≤ 1000 K and the red symbols highlight the large number of points from measurements on Hg, which is often used as a reference material due to availability in high purity and that fact that it is already liquid at room temperature.

**FIG. 19.**
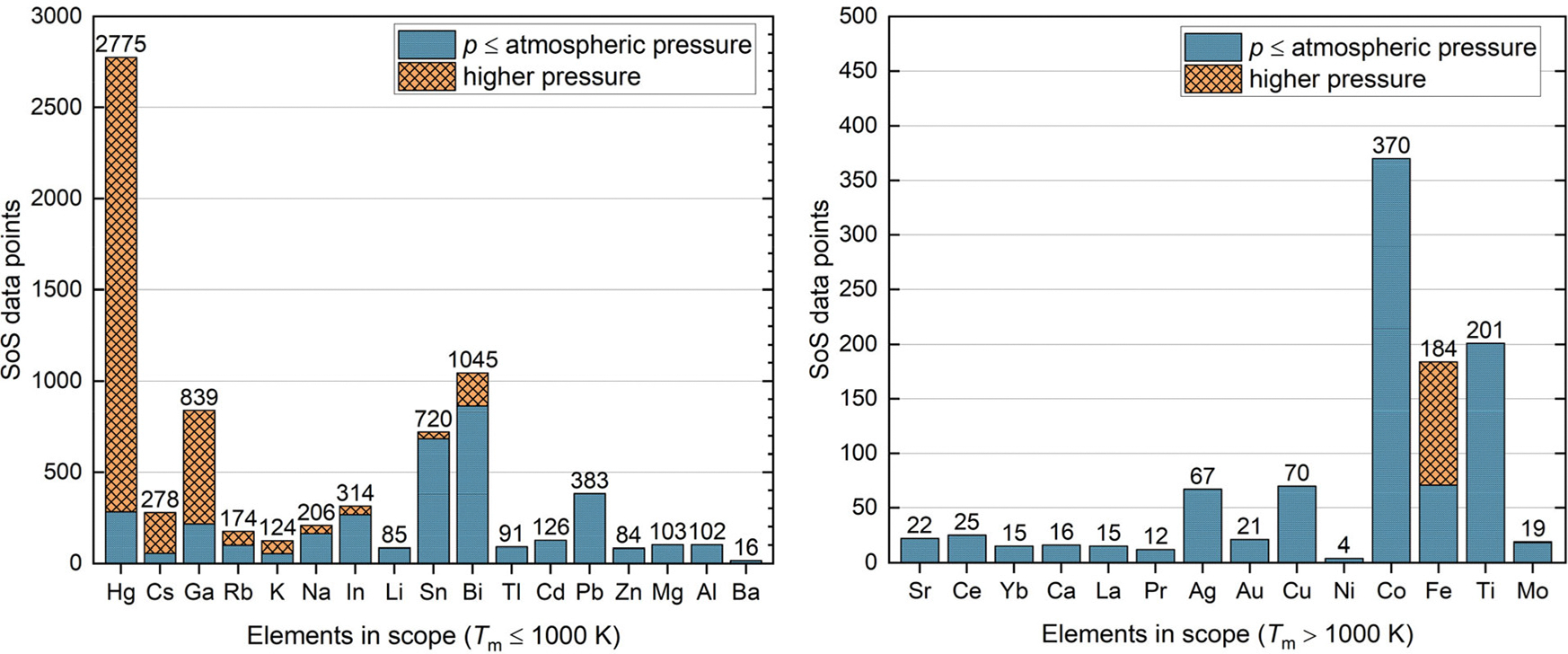
Plot of number of data points for speed of sound vs temperature for pure elements. Elements in scope (left) and elements with melting point >1000 K (right). Elements with zero or one data point are omitted for clarity—see Table S1 in supplementary material for full list.

A graphical summary of speed of sound data for pure elements is given in Fig. 19.

For most elements only a few dozen data points have been reported and low melting elements like Sn or Bi are often used as first material to test or verify a new instrument at atmospheric pressure. That explains why more data are captured for those elements. When analyzing the published results for above-atmospheric pressure two elements stand out. The disproportional amount of data for mercury was already explained. The low melting point, readily and inexpensive availability of high purity samples, and insensitivity to oxidation and other contamination effects make it historically a prime candidate for calibration and test measurements. This trend continues with another element with low melting temperature, gallium. The large amount of data for iron at high pressures stems from geological research interest to learn more about the earth’s inner core. To simulate this high pressure and high temperature environment with iron as main element in the core composition many experiments have been designed but often not with the focus to achieve an uncertainty level that high quality EoS’s require.

The lack of experimental methods for high pressure measurements at simultaneously high temperatures is evident from surveying the available data sources. All this is compounded by the fact that articles reporting on more developed high-temperature methods typically present 10 to 50 data points per paper while high pressure manuscripts commonly report just 3 to 15 points due to the increased experimental effort.

### C. Gaps in available data

In the study of materials in extreme environments, there is often an unbalanced reliance on simulated data compared to experimental data, particularly when it comes to material properties like the speed of sound. While simulations provide valuable insights and can capture complex phenomena, they rely on various assumptions, approximations, and input parameters that may introduce uncertainties. Experimental data, on the other hand, offers direct measurements and validation of material properties in real-world conditions. Kattner recently underlined the need for reliable thermodynamic experimental data.^196^ To achieve a more comprehensive understanding, it is essential to combine the strengths of simulations and experiments, ensuring that the simulated data are validated and calibrated against experimental results where available. In an ideal scenario, this can open opportunities to extrapolate properties into regions of material compositions and parameter space that are not accessible with experimental methods. This will enable a more reliable and accurate characterization of material behavior in extreme environments, including the speed of sound, and facilitate advancements in all relevant scientific and engineering applications.

## IV. CORRELATIONS, MODELS, AND SIMULATIONS

Modeling material behavior is omnipresent in everyday engineering, scientific discovery, and efficient material applications. The increased availability of computational capabilities has made modeling readily attainable. The promise of discovery and optimization is offered by models in artificial intelligence (AI) and machine learning (ML) research spheres. Dramatically increased computation power also reduces the need for limiting the number of model parameters and models describe reals systems more accurate than ever before. Those benefits have spurred considerable scale efforts and programs like the Materials Genome Initiative (MGI) to enable such discovery and optimization through organizing and making the published data easily accessible. Nevertheless, one must remember that the cornerstone models and simulations rest upon empirical data, which rest upon rigorous metrology practices. As outlined in the 2019 National Academies of Sciences, Engineering, and Medicine (NASEM) workshop, “Data-Driven Modeling for Additive Manufacturing of Metals”, transformative advances in manufacturing could be significantly accelerated with the development of models that predict material behavior.^197^ An equation of state is an example of such a model, but an EoS can only be created if accurate material properties are known. Realizing the necessity of empirical data for theoretical models also underscores the need of instruments to capture the data, as outlined for thermophysics by the European Union in 2015.^198^ The need for models was outlined in a recent review by Barrachlin.^199^

This section describes how the speed of sound experimental techniques and the data available from measurements have been applied to theoretical investigations. First, the derivation of the speed of sound into other thermodynamic material properties will be described. It is followed by a review on advancements in how the speed of sound measurements fits into the EoS creation of condensed matter at extreme conditions. Finally, the section will conclude with a brief overview of other models and simulation tools rely on speed of sound measurements. This final part includes a discussion on CALPHAD and DFT, AI, and ML, and computational fluid dynamics and multiphysics models.

In one of P. W. Bridgeman’s seminal papers, 10 fundamental thermodynamic quantities were outlined and it included examples of how thermodynamic material properties are interrelated through mathematical derivation.^200^ The SoS stands out as one of the most captivating properties, primarily due to the multiple derivatives of the Helmholtz energy involved in its computation and the remarkably low uncertainties achievable in its measurement. To put SoS into perspective of other thermodynamic properties, heat capacity offers valuable insights into an EoS fit’s quality, and at least one heat capacity measurement near the triple point is essential to establish the thermodynamic surface accurately if one is basing EoS only on SoS data. Yet, extensive measurements of heat capacities are currently not as beneficial, especially for pure fluids due to the significant measurement uncertainties.

A material’s speed of sound (c), is defined as the square root of the change in pressure (P) over the change in density (ρ) at constant entropy (S), as shown in the following equation:

(1) $$c=\sqrt{{\left(\frac{\partial P}{\partial \rho }\right)}_{s}}.$$

As entropy is not experimentally measured, it is helpful to put c in terms of T,P, and ρ. Putting c into Gibbs thermodynamic potential terms, Z=E+PV-TS, where E is the internal energy of a substance yields the following equation so that T and P are constant:

(2) $$\varepsilon^{2}={\left[{\left(\frac{\partial \rho }{\partial P}\right)}_{T}-\left(\frac{T}{\rho^{2}c_{P}}\right){\left(\frac{\partial \rho }{\partial T}\right)}_{P}\right]}^{-1}.$$

Similarly, putting c into Helmholtz thermodynamic potential terms, Ψ=E-TS, yields the following equation so that T and ρ are constant:

(3) $$c^{2}={\left[{\left(\frac{\partial P}{\partial \rho }\right)}_{T}+\left(\frac{T}{\rho^{2}c_{V}}\right){\left(\frac{\partial \rho }{\partial T}\right)}_{\rho }\right]}^{-1}.$$

As speed of sound is a second-derivative property, density, isobaric heat capacity, $c_{p}$, and isobaric expansivity, α, can be derived with Eqs. (4) and (5) (where specific volume, $v=\rho^{-1}$), and (6), respectively:

(4) $${\left(\frac{\partial \rho }{\partial p}\right)}_{T}=-\frac{1}{c^{2}}+\frac{T\alpha^{2}}{c_{P}},$$

(5) $${\left(\frac{\partial c_{P}}{\partial p}\right)}_{T}=-T{\left(\partial^{2}v/\partial T^{2}\right)}_{P},$$

(6) $$\alpha =-\rho^{-1}(\partial \rho /\partial T{)}_{P}.$$

SoS also relates to other material properties, namely, the Grüneisen parameter, $\gamma_{G},\gamma_{G}=\frac{\alpha c^{2}}{c_{p}}$, adiabatic and isothermal bulk modulus, $B_{S},\;B_{S}=\rho c^{2}$, and compressibility, $K_{S},K_{S}=\frac{1}{B_{S}}$.

### A. Theoretical correlations

One of the most well-known correlations for high pressure data, especially for shock-wave data from shock-compression experiments, is the Hugoniot curve. The Hugoniot curve correlation is a valuable tool for analyzing high-pressure data in materials science and shock physics. By establishing correlations between shock velocity and particle velocity the Hugoniot curve enables the determination of compressibility, shock wave strength, and phase transitions. Thus, the Hugoniot curve [(Eq. (7)] plays a critical role in the study of shock-induced phenomena, material characterization, and the design of materials for applications in extreme environments where U is the shock velocity, and u is the particle velocity, and a and b are positive constants characteristics of the materials and the initial state.

(7) U=a+bu.

This linear relation is equivalent to one of pressure and specific volume and Ruoff showed that coefficients, a, the lattice constant and b, the Burgers’ vector, where $K^{s}$ is the isentropic bulk modulus and $\frac{dK^{s}}{\;dp}$ is the pressure derivative of the isentropic bulk modulus.

(8) $$a={\left(\frac{K^{s}}{\rho^{+}}\right)}^{\frac{1}{2}},\;b=\frac{1}{4}\left[1+\frac{dK^{s}}{\;dP}\right].$$

The initial state is defined with the value of a, which is identified with the isentropic bulk speed of sound.^201^ A review by Graham provides details on the theory and extensive derivations^168^ and Fritz *et al.* provide a good explanation of Hugoniot curves and their and relation to EoS.^202^

### B. Thermodynamic phase diagrams: CALPHAD, equation of state (EoS)

Thermodynamic phase diagrams are crucial tools in understanding the behavior of materials under different temperature and pressure conditions. They provide a visual representation of the stable phases and their relationships, allowing scientists and engineers to make informed decisions about material selection, processing, and applications. These diagrams enable the identification of phase transitions, such as solid–liquid or liquid–gas transformations, and provide insights into the thermodynamic properties, such as melting points, solubilities, and phase equilibria. By utilizing thermodynamic phase diagrams, researchers can optimize processes, predict material behavior, and design materials with specific properties, leading to advancements in various fields, including materials science, chemistry, and engineering.

The CALPHAD (CALculation of PHAse Diagrams) approach and the EoS approach are two distinct methods for constructing thermodynamic phase diagrams. The CALPHAD approach focuses on incorporating experimental data, such as phase equilibria and thermodynamic properties, into a thermodynamic database. It utilizes mathematical models and optimization techniques to fit the data and generate predictive phase diagrams. CALPHAD allows for the description of complex multicomponent systems and provides a comprehensive understanding of phase stability and transformations. On the other hand, the EoS approach is based on the mathematical representation of the relationship between thermodynamic properties, such as pressure, temperature, and volume, using an equation. The best-known EoS is the ideal gas law, PV=nRT. The simplest EoS approach focuses on capturing the bulk properties of materials and is particularly useful for studying simple systems where interactions between individual atoms or molecules dominate. EoS models are often employed to calculate thermodynamic properties and predict phase behavior. While both approaches provide insights into phase stability and transformations, they differ in terms of their underlying principles and applications. The CALPHAD approach is well-suited for complex multicomponent systems, where experimental data are available, enabling accurate predictions of phase diagrams. On the other hand, the EoS approach is more commonly used for simpler systems, where equations can be formulated to describe the thermodynamic behavior of the material. Both approaches contribute to our understanding of materials and play important roles in various fields, including materials science, metallurgy, and geology.

In order to represent the properties of a real fluid, the current state of the art is to express the Helmholtz energy of a fluid as a function of temperature and density, and then to obtain all other thermodynamic properties from mathematical thermodynamic relationships. These Helmholtz-based EoS are the backbone of the highest fidelity digital models of fluids in the chemical process industry. These models are so vital to safe and successful design in the chemical process industry that they have been integrated into widely used commercial simulation software such as Ansys Fluent^203^ and Aspen.^204^ Furthermore, EoS’s are used in materials discovery through machine learning as initiated by the Materials Genome Initiative.^205^ One example is lead and the lead-bismuth eutectic, created out of efforts started in the 1950s and last updated in 2008, to use the liquid metal as a coolant for nuclear reactors.^206^ These use a relatively simple EoS form and cannot accurately compute all properties. Lead’s EoS in the liquid phase has uncertainties of 12% or greater and does not depend on pressure. The Sobolev, Schuurmans, and Benamati EoS has been used to guide simulations.^207^ The lack of data for lead was noticed by IUPAC as a need in 2008, but no notable work has been undertaken to this point. In discussions about the EoS for condensed materials under high pressure, the term “Hugoniot EoS” frequently emerges. Essentially, this term signifies that the EoS was formulated using shock compression experiments as a reference, facilitating extrapolation into the GPa range. To make EoS calculations for detonation states, a JCZS3 database was published in 2018 by researchers at Sandia National Labratory.^208^ As with EoS applicable to more mild ranges (<100 GPa), the lack of data and large uncertainties between investigators leads to difficulties in creating equation of states as noted by Zhang *et al.* for the case of boron carbide (B_4_C).^209^ In 2019, Weck *et al.* published an EoS of bulk niobium from DFT and used the NIST-JANAF thermochemical data to validate molar heat capacity of the EoS.^210^ The NIST-JANAF thermochemical data are a standard reference data (SRD)—SRD13, last updated for data in 1985.^211^

### C. Simulated data and models: MD, DFT, AI, ML

In recent years, the field of condensed matter physics has witnessed remarkable advancements in the generation and utilization of simulated data, revolutionizing our understanding of complex materials and their thermodynamic properties. This section provides a brief overview of the state-of-the-art in simulated speed of sound data for condensed matter physics, highlighting key methodologies such as molecular dynamics (MD) as well as emerging techniques rooted in artificial intelligence (AI) and machine learning (ML). By employing these powerful computational tools, researchers are now able to simulate and explore the behavior of materials at the atomic and molecular level with unprecedented accuracy and efficiency.

The listed methods are distinct, yet complementary approaches used in the field of computational materials science. Molecular dynamics simulations involve the numerical integration of equations of motion to study the behavior of atoms and molecules over time. They provide detailed information on the dynamics and interactions of particles, enabling the investigation of complex systems and processes at the atomic scale. Density functional theory (DFT) is a quantum mechanical approach that utilizes the electron density as a fundamental variable. It enables the calculation of electronic properties and energies, providing insights into the ground-state properties of materials. DFT is widely used for studying the electronic structure, energetics, and thermodynamics of materials. The practice of employing MD involves simulations using either empirical potentials (EP) or DFT. Artificial intelligence and machine learning techniques have gained significant attention in recent years. Their algorithms can be trained to recognize patterns, make predictions by extrapolation, and classify data without explicit programming. In materials science, these techniques are applied to various tasks, such as predicting material properties, discovering new materials, and optimizing experimental and computational workflows. While MD simulations provide dynamic information, DFT offers electronic structure insights, and AI/ML techniques excel in pattern recognition and prediction. Combining these approaches can enhance our understanding of materials by leveraging their respective strengths. For instance, DFT calculations can provide input data for ML models to accelerate the discovery of new materials or improve accuracy in predicting material properties. Molecular dynamics simulations can be integrated with AI/ML methods to simulate and analyze complex material processes with greater efficiency and accuracy. Together, these approaches contribute to advancing our understanding and design of materials for various applications.

MD simulations are used to study transient atomic and molecular physical movements. Commonly, MD simulations rely on material properties from experiments or DFT calculations. An example of how simulations rely on experimental data can be found in Zhang *et al.* using shock compression data of tantalum from Akin *et al.*^182^ to perform *ab initio* MD simulations to predict the melting curve.^212^ Using sound speed measurements has been key to guiding researchers MD simulations: in particular the data collection of Los Alamos Scientific Laboratory (LASL) Shock Hugoniot Data from 1979 is a treasure trove.^213^ An example of this by Ravelo *et al.* reported MD simulations for single crystal tantalum in 2013 up to 300 GPa to study the material’s plasticity.^214^ A 2019 report by Hahn and Fensin, also investigated defects in a tantalum single crystal, used the LASL Shock Hugoniot Data to guide simulations, and suggested that additional data could be very valuable.^215^ Similar MD simulations to that of Hahn and Fensin were reported in 2021 for Cu by Ma *et al.*,^216^ Bryukhanov,^217^ and Schörner.^218^ It should be noted that most experimental data guiding these simulations were published in the 1980s–1990s, and researchers like Ma *et al.* explicitly state in publications that more data are needed to enable a direct comparison with MD results.

In addition to limited data for comparison with MD simulations, some researchers have transitioned to exploring the possibilities of simulated data to fill in the gaps in measurements. In 2022 Bouchet *et al.* used *ab initio* calculations based on work by Hellman *et al.* of Linköping University to calculate the solid thermodynamic properties of iron, namely, SoS.^219^ In 2022, Schörner *et al.* completed DFT-MD simulations on copper to extract the adiabatic SoS, which was then compared to 9 measurements from 620 GPa ± 5.5 GPa to 1130 GPa ± 13.9 GPa at atmospheric temperature that McCoy *et al.* at Sandia National Laboratories reported in 2017.^218,220^ These examples of simulations and experiments are encouraging, but rare, and in the overall review of literature we found that the contemporary body of literature reveals a concerning limitation in the availability of experimental data for condensed matter materials subjected to extreme environments. This scarcity or empirical data under extreme conditions can be attributed to a myriad of challenges: the inherent difficulties in maintaining and manipulating such environments, potential risks associated with conducting these experiments, and the logistical and financial barriers to acquiring appropriate equipment. As a result, researchers often resort to data modeling as a surrogate to fill this void. However, while modeling can provide valuable insights, the sheer volume of accurate data required for the robust application of statistical methods, particularly in training predictive models, is lacking. This paucity of data can sometimes tempt scientists to employ data augmentation techniques to artificially inflate their datasets. Yet, this practice can be perilous. Augmented data, if not carefully curated, can introduce noise, inaccuracies, or biases that may skew findings, leading to erroneous conclusions or predictions. Thus, while the lure of a larger dataset can be enticing, the integrity and authenticity of data remain paramount, especially in a domain as nuanced as condensed matter materials under extreme conditions.

## V. ONE THERMODYNAMIC PROPERTY, MANY APPLICATIONS

This section discusses some current applications for SoS across different industry sectors. Through innovations in speed of sound metrology that increase data availability advances in data-driven theoretical models and applications have been made possible. Applications include the energy industry, particularly in the geothermal and fusion sector, metal additive manufacturing, next-generation semiconductor manufacturing, and defense applications. Additionally, fundamental scientific questions centered around understanding the thermodynamic properties of condensed matter in extreme environments are being explored with direct implications for geophysics. The references selected reflect recent publications that put into context the importance and relevance of the topic covered. The publications reviewed here also represent the current state-of-the-art experimental and theoretical methods for SoS in condensed matter at extreme environments. It should be noted that great effort has been made to direct the reader to publications from reputable sources and contextualized the state-of-the-art without being overbearing with historical context.

Materials with exceptional properties under extreme conditions are used in advanced manufacturing processes. For example, high-temperature alloys and ceramics are employed in aerospace component fabrication, additive manufacturing of metal parts, and high-precision machining applications. Additive manufacturing (AM) of metals has the potential to fundamentally alter the landscape of many important industries, including the aerospace, defense, energy, and medical sectors.^221,222^ It offers unique opportunities to investigate fundamental aspects of material properties as they pertain to data-driven manufacturing, including the deterministic control of material structure-property relationships under stringent manufacturing constraints.^223^ Speed of sound has been used to analyze metal AM parts during and after fabrication.^224^ With this method Thomas *et al.* evaluated differences in manufacturing methods and no material property differences between the traditionally forged and AM manufactured parts were reported.^225^ The lack of understanding the solid to liquid phase transition in metal AM process recently led Yan *et al.* to fabricate a version of the pulse-echo ultrasonic interferometric technique discussed in Sec. II C 1 to monitor in real time the melting process with the first proof of concept metal being gallium for the ultimate application in additive manufacturing.^226^

Accurate properties for liquid metals are essential as AM depends on melting solid materials to the liquid state with subsequent re-solidification. At present, there is a scarcity of information regarding liquid metals, and the limited available data suffers from significant uncertainties that hinder their practical applicability.^79^ It is important to recognize that this lack of accurate data extends to widely utilized pure metallic elements like aluminum and nickel, as well as various alloys such as bronze and stainless steel.^189^ This knowledge gap stalls AM innovation across government, academic, and industrial organizations.^227^ Weirather *et al.* explained that the speed of sound is chosen to ensure a desirable limit of density variation in these studies.^228^ A review on multi-scale and multi-physics metal AM was recently published by Bayat *et al.* which the reader is directed to for additional information.^229^

Materials that can withstand high pressures are critical for high-pressure physics research. They enable the study of exotic states of matter, phase transitions, and the behavior of materials under extreme compression. The material property speed of sound plays a critical role in geology for both Earth and extraterrestrial planetary exploration. This research can provide insights into fundamental physics and help understand planetary interiors. On Earth, it assists geologists in understanding the subsurface structure, composition, and physical properties of geological materials. By measuring the speed of sound in rocks and sediments, geologists can infer valuable information about their density, elastic moduli, and porosity.^230^ This knowledge helps in characterizing rock formations, identifying geological boundaries, and assessing the potential for natural resource exploration. In extraterrestrial planetary exploration, the speed of sound is utilized to study and analyze the composition and structure of celestial bodies. Speed of sound data in planetary exploration missions, for instance, on Mars, can aid in characterizing the properties of the Martian regolith, determining the subsurface composition, and identifying potential water ice deposits so that scientists can gain insights into their density, composition, and potential for supporting life.^231,232^ On moons such as Europa or Enceladus, the speed of sound can provide information about the composition and potential presence of subsurface oceans.^233^ Understanding the speed of sound material property is essential for seismic investigations. Seismic data, combined with speed of sound measurements, can help scientists interpret seismic waves, identify subsurface structures, and determine the presence of geological features such as faults, volcanoes, or impact craters.^234^ This knowledge is crucial for reconstructing the geological history and evolution of planets, moons, and asteroids, as well as assessing their potential for habitability or resource exploration. In summary, the use of the speed of sound in geology enables geologists to study Earth’s subsurface and gain insights into the composition, structure, and resource potential of geological materials. By utilizing this property, scientists can deepen their understanding of Earth and expand our knowledge of the solar system, ultimately paving the way for future exploration and discoveries.

Looking at materials at extreme conditions from an energy standpoint, nuclear power plants require materials that can withstand extreme temperatures, pressures, radiation, and corrosive environments.^235,236^ These materials ensure the safety and efficiency of nuclear reactors and their components, including fuel cladding, reactor vessels, and control rods. In fusion energy research, understanding the speed of sound in plasma and its interactions with surrounding materials is critical for achieving and maintaining controlled fusion reactions. By measuring the speed of sound in plasma, scientists can assess the compressibility and propagation characteristics of plasma waves, aiding in the design and optimization of fusion reactors.^237^ This knowledge contributes to the development of more efficient and stable fusion processes, which have the potential to revolutionize the future of clean energy production. In addition to nuclear power plants, geothermal energy harnesses heat from deep within the earth. Materials capable of withstanding high temperatures and corrosive environments are essential for geothermal power plants, including heat exchangers, turbines, and drilling equipment.

Materials that can withstand high temperatures, pressures, and harsh environments are crucial for aerospace and defense applications. These include heat-resistant alloys for jet engines, ceramic matrix composites for hypersonic vehicles, and materials for armor and protective gear. By studying the speed of sound in materials used for missile construction, engineers can assess their strength, elasticity, and resistance to high-speed impacts.^238^ This information is crucial in designing missile components that can withstand extreme conditions and maintain structural integrity during flight and beyond. By measuring the speed of sound in materials used for armor, such as metals or composite materials, engineers can evaluate their ability to withstand ballistic impacts and dissipate kinetic energy.^239^ This knowledge aids in the design of protective armor systems that offer enhanced durability, resistance to penetration, and overall ballistic performance. Overall, the use of the speed of sound material property enables researchers and engineers to make informed decisions in their pursuit of efficient fusion energy generation and the design of robust defense systems, contributing to advancement in both fields and ensuring the safety and security of energy and defense applications.

**FIG. 20.**
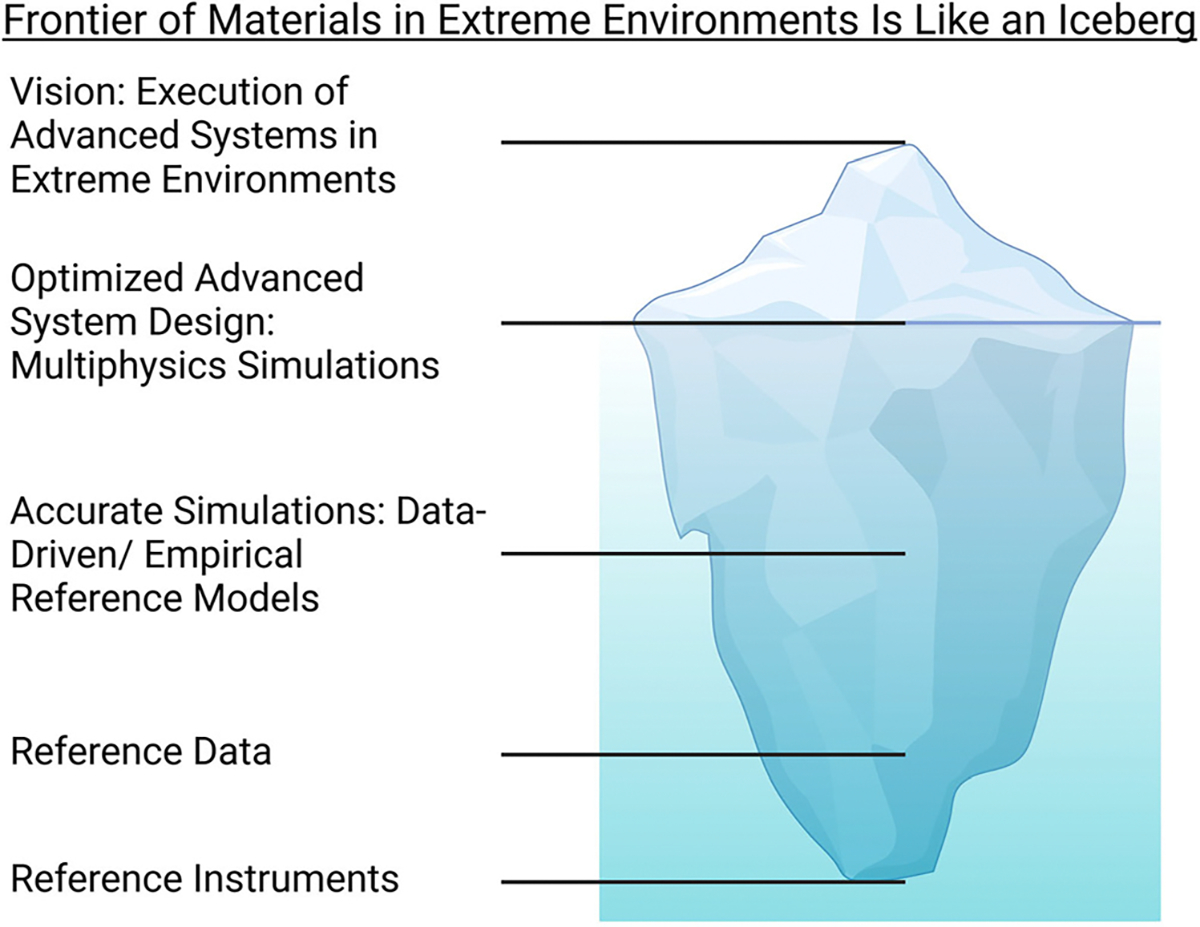
Understanding the impact of materials in extreme environments.

The utilization of liquid metals in various advanced technologies has become increasingly prominent. The increasing focus on incorporating liquid phase metals should underscore the importance of investigating thermodynamic materials properties promptly (Fig. 20). This approach will enable innovation based on a solid understanding of fundamental principles, guiding optimized design instead of relying on the trial-and-error approach often associated with Edisonian methods.

### A. Future directions

In the culmination of this review on the speed of sound for materials under extreme conditions, several future directions have been identified, spanning across three paramount areas.

Concerning measurement uncertainty, two critical pathways are evident:

1. The establishment of a unified uncertainty analysis that can serve as a benchmark for all subsequent evaluations, thus reducing ambiguity.
2. The training, execution, and accountability of a comprehensive framework for measurement reporting, including uncertainty, which would allow for a more standardized assessment of potential variances during measurements.
  Within the realm of measurement methods:
3. Emphasis should be placed on the creation of new measurement instruments that are built on modern electronics and controls that can be automated potentially leveraging advancements in other scientific domains to refine the accuracy and reliability of measurements to generate reference quality data for materials under extreme conditions.
4. A more profound exploration of pure elements is essential. From this foundation, we can enhance our grasp of multicomponent, or alloy, materials.
  Lastly, in the context of theoretical models and simulations:
5. The pursuit of repeatability and well-defined models, coupled with a comprehensive acknowledgment of simulation limitations, aims to offer a more intricate and foresightful understanding of material behavior under critical conditions. Such endeavors offer insights indispensable for forthcoming investigations.

In conclusion, the speed of sound material property offers opportunities to characterize other properties through thermodynamic integration, perform measurements with lower uncertainty and more automated compared to other material properties. From these metrology advantages, the impact of new rigorous speed of sound data can span many industries. Ensuring that the speed of sound for materials under extreme conditions is accurately recorded is not just distant scholarly endeavor, but a critical component for a successful and safe future.

## Supplementary Material

Supp1

SUPPLEMENTARY MATERIAL

See the supplementary material Document containing a list of the naming schemes for different terms plus Table S1 containing the number of data points for sound speed vs temperature for pure compounds in scope for this manuscript.

## Figures and Tables

**TABLE I.** Spectroscopic methods for measuring sound speed of a condensed matter material in a DAC.

Spectroscopic method	Principle	Experimental technique	Notes

Brillouin scattering	Interaction between light and acoustic phonons leading to a frequency shift in a material	A laser beam is focused onto the sample in the DAC, and the scattered light is analyzed to measure the frequency or Brillouin shift. The shift provides information about the elastic properties, speed of sound, and compressibility of the material.	Experiments are limited to transparent samples
Inelastic x-ray scattering (IXS)	The electronic and lattice vibrations (phonons) excitations in a material	High-energy x-rays from a synchrotron source are scattered by the sample within the DAC. The energy and momentum transfer of the scattered x-rays are measured to extract information about electronic band structures, material property correlations, and phonon properties.	Well established method, requires large synchrotron instrumentation
Picosecond acoustics (PA)	Generation and detection of ultrafast acoustic waves (phonons) in materials using femtosecond laser pulses	A femtosecond laser generates acoustic waves in the sample, and their propagation and behavior are probed using ultrafast laser spectroscopy. Changes in the optical properties of the material caused by the acoustic waves are detected.	Newer method, accessible, simultaneous determination of sample length and travel time of the sound wave

**TABLE II.** Outline of some recent shock wave experiments including the material studied, the number of measurements reported, the pressure and temperature parameters, and year published.

Material	Number of measurements	Pressure (GPa)	Year	Note	Reference

	9	5–22	2015		180
Lithium fluoride (LiF)	4	134 and 152	2021	Work disagreed with prior melting curve^180^	181
	13	118–231	2023		73
Bismuth (Bi)	6	2.2–4.5 for liquid; 8–14 for solid	2015	Demonstrating ability to study difference between solid and liquid phase materials with shock compression	171
Scandium (Sc)	6	19.5–82	2017	Also reported measurements of uncompressed Sc to compare; uncertainty in shock speed was estimated at ~4% and pressure ~7% by propagating through the Rankine-Hugoniot equations	173
Tin (Sn)	3	52–84	2019		172
Tantalum (Ta)	14	37–363	2019		182
	51	70–2247	2023		183
Cerium (Ce)	17	7–25	2020		178
	13	8.4–23.5	2021		179
Silver (Ag)	10	60–300	2021	Uncertainties determined using the Monte Carlo method^184^	181
Magnesium (Mg)	5	310–1317	2022	Did not take into consideration pressure uncertainties into overall measurement uncertainty believing to be negligible; but noted −14 to +70 GPa on Be stresses in SI	185
Copper (Cu)	8	117–280	2022		186
Iron (Fe)	6	60–1400	2018	Reported pressure uncertainty of 26 GPa (1 σ) for the high pressure region	132

**TABLE III.** Examples of available databases—this list is not exhaustive and not in a particular order.

Database name	Lead country	Description

Materials Project (materialsproject.org)	USA	Open web-based access to computed information on known and predicted materials
Nomad materials data (nomad-lab.eu)	Germany	Input and output files of all important computational materials science codes
MatNavi (NIMS Material Databases) (mits.nims.go.jp)	Japan	A set of databases for Thermophysical Properties (density, surface tension, viscosity of metals), computational electronic structures, computational phase diagrams, crystal structures and x-ray diffraction information, diffusion data, a polymer database and more
AFLOW (www.aflowlib.org)	USA	Globally available database containing inorganic materials with calculated properties through high-throughput calculations
Computational Materials Repository (cmr.fysik.dtu.dk)	Sweden/ Denmark	Infrastructure to enable collection, storage, retrieval, and analysis of data from electronic-structure codes
Open Quantum Materials Database (www.oqmd.org)	USA	Database of DFT-calculated thermodynamic and structural properties of inorganic materials
2D Material Encyclopedia (www.2dmatpedia.org)	Singapore	Computed properties of 2D materials obtained by exfoliation of existing layered materials and chemical substitution from 2D materials
Materials Cloud (www.materialscloud.org)	Europe	Collaborative open science platform offering educational, research, and archiving tools; simulation software and services; curated and raw data
JARVIS (Joint Automated Repository for Various Integrated Simulations) (jarvis.nist.gov)	USA	Repository designed to automate materials discovery and optimization using classical force-field, density functional theory, machine learning calculations and experiments
NIST Alloy Data (trc.nist.gov/metals_data)	USA	Free curated experimental thermophysical property data from published articles for primary unary, binary, and ternary alloy systems
Shock Wave Database, 2003 (http://teos.ficp.ac.ru/rusbank)	Russia	Collection of numerous shock-wave experimental points including measurements of thermodynamic properties of matter in shock and isentropic release waves. Collected are ca. 15 000 points from 300 references for 500 substances

## Data Availability

Data sharing is not applicable to this article as no primary data are presented. All data used for analysis as presented in Sec. III is freely available at: https://trc.nist.gov/MetalsAlloyUI/

## NOMENCLATURE

*A*: Ampere
AI: Artificial intelligence
AM: Additive manufacturing
*a*: Equation constant
*b*: Equation constant
*B*_*s*_: Bulk modulus
CALPHAD: Calculation of phase diagrams
CW: Continuous wave
CCT WG-NCTh: Consultative Committee for Thermometry’s Non-Contact Thermometry Working Group
*c*: Speed of sound
*c*_*p*_: Specific heat at constant pressure
*c*_*v*_: Specific heat at constant volume
DAC: Diamond anvil cell
DFT: Density functional theory
ETH: Eidgenössische Technische Hochschule
EoS: Equation of state
*E*: Internal energy
GUM: Guide to the expression of uncertainty in measurement
h-BN: Hexagonal boron nitride
HP-CAT: High-pressure collaborative access team
HPHT: High pressure high temperature
IEC: International Electrotechnical Commission
IEX: Isobaric expansivity expansion
IPPS: International practical pressure scale
IR: Infrared
ISO: International Organization for Standardization
ITS: International temperature scale
IXS: Inelastic x-ray scattering
JANAF: Joined Army-Navy-Air Force
JCGM: Joint Committee for Guides in Metrology
*K* ^*s*^: Compressibility
LASL: Los Alamos Scientific Laboratory
LCLS: Linac coherent light source
LINAC: Linear accelerator
LU: Laser ultrasonic
LH: Laser heating
LVP: Large volume press
MD: Molecular dynamics
MGI: Materials genome initiative
ML: Machine learning
NASEM: National Academies of Science, Engineering, and Medicine
NIST: National Institute of Standards and Technology
NPD: Nano-polycrystalline diamond
PA: Picosecond acoustic
PDV: Photo doppler velocimetry
PE: Paris–Edinburgh
PTM: Pressure transmitting medium
PTZ: Pb(Zr-Ti)O3 – lead zirconate titanate
*P*: Pressure
*P*_*c*_: Critical pressure
*T*: Absolute temperature
*T*_*c*_: Critical temperature
*T*_*m*_: Melting temperature
SACLA: Spring-8 Angstrom Compact Free Electron Laser
SC: Shock compression
SNCSC: Springer Nature Customer Service Center GmbH
SoS: Speed of sound
SRD: Standard reference data
*S*: Entropy
s: Second
TC: Thermocouple
TDBS: Time domain Brillouin scattering
*V*: Volume
u^−^: Particle velocity
U_T_: Uncertainty in temperature
U_P_: Uncertainty in pressure
U_c_: Combined uncertainty
U: Shock velocity
VISAR: Velocity interferometer system for any reflector
WC: Tungsten carbide
XAS: X-ray absorption spectroscopy
XRD: X-ray diffraction analysis
XFEL: X-ray free-electron laser facility
*α*: Isobaric expansivity
Δ: Change
𝑣: Specific volume
*ρ*: Density
