# Supplementary material for: Speed of sound for understanding metals in extreme environments
Source: Appl Phys Rev. Author manuscript; Available in PMC 2026 Jul 30. (PMC13417931; doi:10.1063/5.0186669)
Supplement: Supp1 [file NIHMS2028954-supplement-Supp1.zip › SI_Table_S1.pdf]

Table for SI: Number of data points for Sound speed vs. temperature for pure compounds in scope.

Element	Melting point, K	# of data points	*p* £ atmospheric pressure	higher pressure
Hg	234.32	2775	282	2494
Fr	294.15	0	0	0
Cs	301.65	278	57	221
Ga	302.91	839	215	624
Rb	312.45	174	99	75
K	336.65	124	56	68
Na	370.94	206	160	46
In	429.75	314	267	47
Li	453.65	85	85	0
Sn	505.08	720	684	36
Bi	544.55	1045	864	182
Tl	577.15	91	91	0
Cd	594.22	126	126	0
Pb	600.61	383	383	0
Zn	692.68	84	84	0
Mg	923.15	103	103	0
Al	933.47	102	102	0
Ra	969.15	0	0	0
Ba	1000.15	16	16	0
Sr	1050.15	22	22	0
Ce	1072.15	25	25	0
Yb	1097.15	15	15	0
Eu	1095.15	0	0	0
Ca	1115.15	16	16	0
La	1193.15	15	15	0
Pr	1204.15	12	12	0
Ag	1234.93	67	67	0
Nd	1289.15	1	1	0
Ac	1323.15	0	0	0
Au	1337.33	21	21	0
Sm	1345.15	1	1	0
Cu	1357.77	70	70	0
Pm	1315.15	0	0	0
U	1408.15	0	0	0
Mn	1519.15	0	0	0
Be	1560.15	0	0	0
Gd	1586.15	1	1	0
Tb	1632.15	1	1	0
Dy	1685.15	1	1	0

Ni	1728.15	4	4	0
Ho	1745.15	1	1	0
Co	1768.15	370	370	0
Er	1802.15	1	1	0
Y	1795.15	1	1	0
Fe	1811.15	184	71	113
Sc	1814.15	0	0	0
Tm	1818.15	0	0	0
Pd	1827.95	1	1	0
Pa	1845.15	0	0	0
Lu	1936.15	0	0	0
Ti	1943.15	201	201	0
Th	2023.15	0	0	0
Pt	2041.35	1	1	0
Zr	2127.15	0	0	0
Cr	2180.15	1	1	0
V	2183.15	1	1	0
Rh	2236.15	0	0	0
Tc	2430.15	0	0	0
Hf	2506.15	0	0	0
Ru	2606.15	0	0	0
Ir	2719.15	0	0	0
Nb	2750.15	0	0	0
Mo	2895.15	19	18	1
Ta	3290.15	1	0	1
Os	3306.15	0	0	0
Re	3458.15	0	0	0
W	3687.15	1	0	1
